# pH-Activated Nanoplatform Derived from M1 Macrophages’ Exosomes for Photodynamic and Ferroptosis Synergistic Therapy to Augment Cancer Immunotherapy

**DOI:** 10.34133/bmr.0153

**Published:** 2025-03-06

**Authors:** Yawen Guo, Ruijie Qian, Xin Wei, Chunwang Yang, Jing Cao, Xiaoming Hou, Xiaokuan Zhang, Tingting Lv, Lu Bai, Daoyu Wei, Rumeng Bi, Baoen Shan, Zhiyu Wang

**Affiliations:** ^1^ Department of Immuno-Oncology, The Fourth Hospital of Hebei Medical University, Shijiazhuang, Hebei 050000, P.R. China.; ^2^ Department of Interventional Radiology, The First Affiliated Hospital of Zhengzhou University, Zhengzhou, Henan 450000, P.R. China.; ^3^ Department of Ultrasound, Beijing Children’s Hospital, Capital Medical University, National Center for Children’s Health, Beijing, P.R. China.

## Abstract

Combining nanomedicine with immunotherapy offers a promising and potent cancer treatment strategy; however, improving the effectiveness of the antitumor immune response remains challenging. A “cold” tumor microenvironment (TME) is a marked factor affecting the efficacy of immunotherapy. Herein, intracellular-acidity-activatable dynamic nanoparticles (NPs) were designed for precision photodynamic immunotherapy and ferroptosis in cancer. M1 macrophage-derived exosomes (Mex) were constructed to coassemble the photosensitizer SR780, Fe^3+^, and the antioxidant enzyme catalase (CAT). By further modifying the RS17 peptides on the NPs, we increased their tumor-targeting capability and blocked the CD47–signal regulatory protein checkpoint, enabling macrophages to effectively phagocytose tumor cells. With proper particle size and dual targeting, including homologous targeting and RS17 targeting, FeSR780@CAT@Mex-RS17 NPs were able to accumulate effectively at the tumor site. These NPs can deliver exogenous CAT to relieve the hypoxic TME and enhance the therapeutic effects of photodynamic therapy. SR780 triggered photodynamic therapy to produce reactive oxygen species and induced immunogenic cell death to release danger-associated molecular patterns. In combination with Fe^2+^-induced ferroptosis, long-term immunotherapeutic effects can be obtained by reprogramming “cold” TMEs into “hot” TMEs. Upon laser irradiation, the designed FeSR780@CAT@Mex-RS17 NPs exert potent antitumor efficacy against both the Lewis lung carcinoma subcutaneous xenograft tumor model and lung orthotopic and liver metastasis models. The NPs suppressed the growth of the primary tumor while inhibiting liver metastasis, thereby exhibiting great potential for antitumor immunotherapy.

## Introduction

The ability of cancer immunotherapy to specifically eliminate tumor cells has revolutionized traditional treatment strategies since its discovery several decades ago [1]. Although immune checkpoint blockade therapy has achieved marked clinical success, it benefits only a small percentage of patients [2]. Tumor biology and cancer immunotherapy have revealed that the tumor microenvironment (TME) plays a vital role in inhibiting cancer immunotherapy through various mechanisms [3]. For instance, hypoxia and slight acidity are notable TME characteristics of solid tumors that can directly hamper the intrinsic cytotoxic function of CD8^+^ T lymphocytes, as well as the function of dendritic cells (DCs) and macrophages, which are the major immune response agents against cancer [4]. Furthermore, they may undermine antitumor immunity by causing the tumor to accumulate suppressive immune cells, cytokines, and metabolites [5]. Therefore, improving the response of solid tumors to cancer immunotherapy is vital.

Over the past several decades, photodynamic therapy (PDT) has become a high-profile method for cancer treatment. PDT requires 3 components: a photosensitizer, light, and oxygen. During treatment, the laser irradiation transfers energy to the photosensitizer. Reactive oxygen species (ROS) are formed when excited photosensitizers transfer energy to the surrounding oxygen molecules, causing cancer cells to undergo apoptosis [6]. PDT provides benefits over traditional chemotherapy, including a reduction in side effects and the prevention of drug resistance [7]. Substantial research has demonstrated the effectiveness of PDT in inducing immunogenic cell death (ICD), which is a promising approach for immune activation [8]. As a result of ICD, antigens and danger-associated molecular patterns (DAMPs) are released onto the cell surface, and factors such as high mobility group box 1 (HMGB1) and adenosine-5′-triphosphate (ATP) are released. Under endoplasmic reticulum stress, calreticulin (CRT), a soluble protein in the endoplasmic reticulum, is translocated to the surface of tumor cells. The binding of CRT to the CD91 receptor facilitates the recognition of tumor-associated antigens by antigen-presenting cells [9]. Toll-like receptor 4 interacts with HMGB1 to facilitate DC maturation and release proinflammatory cytokines to produce potent immune stimulation [10]. Antitumor T cells and cytokines transform the TME and enhance immunotherapy responses by converting “cold” immunosuppressive tumors to “hot” immunoresponsive ones [11].

However, PDT is limited by a hypoxic TME [12]. In PDT, oxygen is consumed to produce ROS, and rapid consumption of oxygen leads to hypoxia, which further impairs the efficiency of PDT. In contrast, preexisting tumor hypoxia and PDT-induced hypoxia activate hypoxia-inducible factor 1α (HIF-1α), which is strongly associated with poor prognosis and tumor progression [13]. Accordingly, alleviating tumor hypoxia is crucial for enhancing the therapeutic efficacy of PDT. Catalase (CAT) can form oxygen in malignant tumors by catalyzing endogenous hydrogen peroxide (H_2_O_2_; ≈50 × 10^−6^ to 100 × 10^−6^ mM), which offers an attractive method for overcoming hypoxia caused by tumors [14]. Enzyme-like activity not only produces oxygen and promotes the polarization of M2 to M1 macrophages but also generates ROS (•OH and ^1^O_2_) and depletes glutathione (GSH) in the TME to expose necrotic cell fragments and reverses the immunosuppressive TME by inducing DC maturation and infiltration of cytotoxic T lymphocytes (CTLs) in tumors [15]. The delivery of exogenous CAT to alleviate tumor hypoxia can be accomplished using different nanoagents. For CAT-triggered hypoxia modulation, efficient encapsulation of CAT, accurate delivery of CAT to tumor sites, and generation of oxygen remain the major challenges [16].

Ferroptosis is a type of cell death recently discovered that is triggered by iron accumulation and lipid peroxidation in cells, and it differs from other types of programmed cell death, such as apoptosis, necrosis, and autophagy [17]. The first step is to reduce the Fe^3+^ stored in tumor cells to Fe^2+^ by consuming GSH [18]. The Fenton reaction produces hydroxyl free radicals (•OH), which damage cell membranes by forming lipid peroxides. Finally, glutathione peroxidase 4 (GPX4) expression is indirectly reduced by GSH reduction, further disrupting intracellular redox homeostasis and enhancing PDT’s therapeutic effects [19–21]. Conventional ferroptosis therapy, as well as other therapies, usually has limited therapeutic effects when combined with no high levels of selectivity or intelligence [13]. Excessive iron accumulation can lead to organ dysfunction [22]. Considering this, it is of important interest to develop a photosensitizer that is coordinated with Fe^3+^ and activated only in the TME with an “on/off” function.

Exosomes are vesicles of 40 to 100 nm in size that are released by various cell types into the microenvironment and mainly contain proteins, RNA, and lipids [23]. Research indicates that macrophage-derived exosomes exhibit immune cytotoxicity against tumor cells and possess the ability to cross physical barriers as well as inherent targeting properties and enhanced biocompatibility. There is great potential for its use as a new drug delivery system, which could open new avenues for tumor immunotherapy. Furthermore, M1 macrophage-derived exosomes (Mex) can inhibit tumor progression by directly promoting tumor cell apoptosis or enhancing tumor immunity [24]. Compared to many other types of nanomedicine delivery platforms, the Mex strategy offers the well-established benefits of exosomes from endogenous cells [25]. Additionally, the DSPE-PEG_2000_-Mal-RS17 peptide (RS17 peptide), a peptide with antitumor properties, targets CD47 in cancer cells, inhibits the CD47–signal regulatory protein alpha (SIRPα) signaling pathway, and increases macrophage engulfment [26]. CD47 is highly expressed in tumor cells, making it an important player in macrophage phagocytosis [27].

The “don’t eat me” signal is released by CD47 in conjunction with its receptor, SIRPα, on macrophages, which weakens macrophage phagocytosis, thereby facilitating tumor cell escape from immune surveillance and clearance [28]. Blocking CD47–SIRPα has demonstrated promising effects in curbing tumor growth by increasing macrophage phagocytosis and stimulating CTL cross-presentation of antigens [27]. Increased phagocytosis plays a critical role in reprogramming the immunosuppressive TME and restoring the balance of the immune responses in this case [29]. Furthermore, because of the innate tumor-homing ability of nanovesicles and the specificity of the RS17 peptide to bind CD47, they displayed good tumor-targeting properties. Therefore, we propose that combining ferroptosis, the ICD effect, and CAT oxygen supply with CD47–SIRPα blockade may be an effective strategy for enhancing macrophage phagocytosis and improving cancer immunotherapy.

**Fig. 1.**
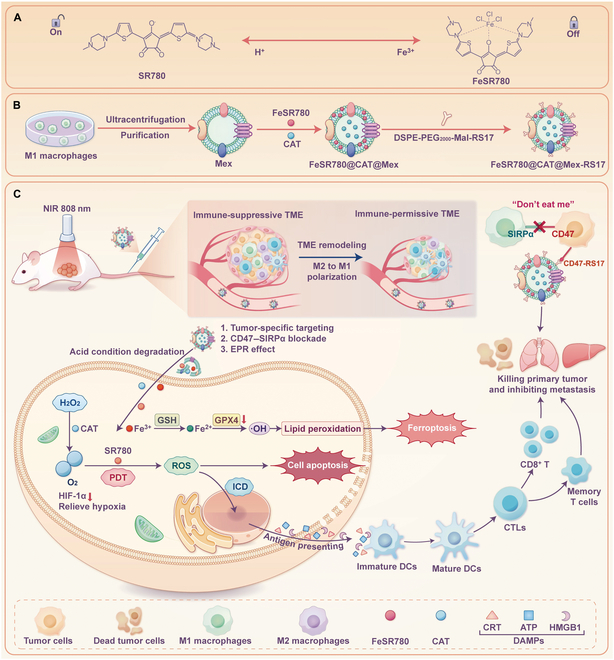
Illustration of FeSR780@CAT@Mex-RS17 nanoparticles (NPs) and their antitumor mechanisms. (A) The structure conversion of SR780 and FeSR780 in acidic and Fe^3+^ environments, respectively. (B) Fabrication of FeSR780@CAT@Mex-RS17 NPs. (C) FeSR780@CAT@Mex-RS17 accumulates at tumor sites owing to (1) tumor-specific targeting, (2) CD47–signal regulatory protein alpha (SIRPα) blockade, and (3) the enhanced permeability and retention (EPR) effect. FeSR780 is activated in the tumor microenvironment (TME). The released Fe^3+^ ions are reduced into Fe^2+^ ions by glutathione (GSH) and induce the death of cells through ferroptosis, the released SR780 produces reactive oxygen species (ROS) under 808-nm laser irradiation, and catalase (CAT) catalyzes the production of oxygen by using hydrogen peroxide to alleviate hypoxia in tumor and enhance photodynamic therapy (PDT). The immune system is activated and elicits potent adaptive antitumor responses that can directly kill tumor cells. Mex, M1 macrophage-derived exosomes; NIR, near infrared; GPX4, glutathione peroxidase 4; HIF-1α, hypoxia-inducible factor 1α; ICD, immunogenic cell death; DC, dendritic cell; CTLs, cytotoxic T lymphocytes; CRT, calreticulin; ATP, adenosine-5′-triphosphate; HMGB1, high mobility group box 1; DAMPs, danger-associated molecular patterns.

In this study, a novel amphiphilic photosensitizer, croconaine dye SR780, was designed and synthesized, which exhibited PDT effects in response to the potential of hydrogen (pH) after excitation with an 808-nm laser. When Fe^3+^ chelated with SR780 (FeSR780), the fluorescence of SR780, the ultraviolet absorbance, and even the PDT effect are abolished, turning to an “off” state. When FeSR780 was exposed to acidic conditions, its characteristics were restored, allowing it to turn on. Mex loaded with CAT and FeSR780 (FeSR780@CAT@Mex) was prepared. Next, FeSR780@CAT@Mex was further modified with the RS17 peptide to form FeSR780@CAT@Mex-RS17 NPs (Fig. 1). Following intravenous administration, FeSR780@CAT@Mex-RS17 accumulated at tumor sites owing to its innate homing ability and affinity for CD47 on tumor cells. Then, an 808-nm laser was used to irradiate the tumor sites. The degradation of FeSR780 under acidic conditions released SR780 and Fe^3+^, and the state changed from “off” to “on” after the coordination bonds were ruptured. Upon exposing SR780 to near-infrared photoirradiation, direct tumor ablation and ICD generation were mediated by photodynamic effects. CAT catalyzes the production of oxygen by using hydrogen peroxide to alleviate hypoxia and enhance PDT. The Fe^3+^ released by FeSR780 was reduced to Fe^2+^ by GSH. The Fenton reaction generates •OH, which increases the level of lipid peroxides and destroys the membrane structure and function. GSH consumption indirectly inhibits GPX4 expression, disrupts the redox balance, and ultimately causes ferroptosis. With the combination treatment modality and the close synergy between each component, the immune system is activated and elicits potent adaptive antitumor responses that can directly kill tumor cells.

## Materials and Methods

### Cell culture and macrophage polarization

The murine lung cancer cell line Lewis lung carcinoma (LLC) and macrophage cell line RAW264.7 were obtained from our laboratory and maintained in Dulbecco’s modified Eagle’s medium (DMEM) supplemented with 10% fetal bovine serum (FBS) and 1% penicillin/streptomycin in a humidified incubator at 37 °C with 5% CO_2_. Bone marrow was isolated and collected from C57BL/6 mice to obtain bone-marrow-derived DCs (BMDCs). BMDCs were cultured in RPMI 1640 medium containing 10% FBS and 200 U/ml mouse granulocyte–macrophage colony-stimulating factor. The morphology and growth status of cells were visualized daily. The cell culture medium was then replaced according to the manufacturer’s instructions. To induce M2 macrophage polarization, RAW264.7 cells (unpolarized, naïve M0 macrophages) were stimulated with 20 ng/ml interleukin-4 (IL-4) for 24 h when they reached 50% to 60% confluence. RAW264.7 cells were incubated with lipopolysaccharide (100 ng/ml) to induce M1 macrophage polarization.

### Exosome extraction and drug loading

Exosome-free FBS was prepared by ultracentrifugation at 120,000 × g overnight at 4 °C, and the supernatant was collected. M1 macrophages were cultured in DMEM supplemented with 10% exosome-free FBS for 48 h. To obtain Mex, DMEM was centrifuged at 800, 3,000, and 10,000 × g for 10, 20, and 30 min, respectively, at 4 °C, followed by filtration through 0.22-μm filters to remove cell debris and large extracellular vesicles. The cell supernatants were then ultracentrifuged at 100,000 × g for 70 min at 4 °C and washed once with phosphate-buffered saline (PBS) to obtain the Mex. Mex pellets were collected and resuspended in PBS. The obtained Mex, CAT, and DSPE-PEG_2000_-FeSR780 were ultrasonicated for 15 min to prepare FeSR780@CAT@Mex. DSPE-PEG_2000_-RS17 (Ruixi, Xi’an, China) and/or DSPE-PEG_2000_-ICG (Ruixi, Xi’an, China) were incubated with FeSR780@CAT@Mex for 30 min at 37 °C to form FeSR780@CAT@Mex-RS17 or ICG-FeSR780@CAT@Mex-RS17, respectively. To remove unreacted reagents and reaction byproducts, the samples were ultrafiltered using centrifugal filter devices (100-kDa molecular weight cutoff). The morphology and structure of FeSR780@CAT@Mex-RS17 were observed by transmission electron microscopy (TEM). Dynamic light scattering (DLS) and zeta potential measurements were performed using Malvern Zetasizer Nano.

### Western blot analysis

The protein expression profiles of FeSR780@CAT@Mex-RS17, Mex, and M1 macrophage cell membranes were measured by Western blot (WB). In brief, different samples were washed thrice with precooled PBS and lysed in radioimmunoprecipitation assay lysis buffer mixed with 10% protease inhibitor. The lysates were collected and centrifuged at 12,000 rpm for 20 min. The protein concentrations in the supernatant were quantified using a bicinchoninic acid protein assay kit. The prepared proteins (20 μg per lane) were separated by 10% sodium dodecyl sulfate polyacrylamide gel electrophoresis and subsequently transferred onto polyvinylidene fluoride membranes. After blocking with 5% skim milk for 1 h, membranes were incubated with different primary antibodies (Table S1) overnight at 4 °C. Subsequently, the polyvinylidene fluoride membranes were washed thrice in Tris-buffered saline with Tween-20 for 5 min each. Membranes were then incubated with secondary antibodies (1:5,000) for 1 h at room temperature. Protein bands were exposed and imaged using an enhanced chemiluminescence kit after repeating the membrane washing procedure.

### Exosome labeling and cellular uptake

LLC cells were cultured in the medium at a concentration of 1 × 10^5^ cells/ml and incubated with indocyanine green (ICG)-labeled FeSR780@CAT@Mex-RS17 overnight. Monoclonal fluorescein isothiocyanate-labeled phalloidin was used to stain the cytoskeleton, followed by staining of the cell nucleus with 4′,6-diamidino-2-phenylindole (DAPI). Confocal laser scanning microscopy (CLSM) was used to measure the intracellular location of ICG-labeled exosomes, which was quantified using flow cytometry (FCM).

### Cell viability test

(a) LLC cells were plated (5 × 10^3^ cells per well) in 96-well plates and allowed to grow overnight. The cells were then treated with PBS, Mex-RS17, CAT@Mex-RS17 (−), CAT@Mex-RS17 (+), FeSR780@Mex-RS17 (−), FeSR780@Mex-RS17 (+), FeSR780@CAT@Mex-RS17 (−), or FeSR780@CAT@Mex-RS17 (+) for 24 h (FeSR780: 2 μg/ml; CAT: 2 μg/ml; (+) indicates irradiation with an 808-nm laser [0.5 W/cm^2^, 0.5 min] and (−) indicates no irradiation). (b) LLC cells, L-02 cells, human umbilical vein endothelial cells, and L929 cells (5 × 10^3^ cells/well) were seeded in 96-well plates. After 12 h of preincubation, various concentrations (1, 5, 10, and 50 μg/ml) of blank Mex nanovesicle solution were added to different cell culture media. (c) Various lung cancer cell lines, including LLC, A549, NCI-H1299, and HCC827 (5 × 10^3^ cells/well), were seeded in 96-well plates and cultured for 12 h. After that, the cells were treated with FeSR780@CAT@Mex-RS17 NPs (+) (FeSR780: 2 μg/ml; CAT: 2 μg/ml; 0.5 W/cm^2^ and 0.5 min). After the indicated treatments, 200 μl of serum-free DMEM containing 20 μl of the Cytotoxicity Assay Kit (Cell Counting Kit-8 [CCK-8]) solution was used to incubate the cells for 1 h. The absorbance was measured at 450 nm.

### Intracellular ROS measurement

To detect the intracellular ROS levels, LLC cells were treated with PBS, CAT@Mex-RS17, FeSR780@Mex-RS17 (+), or FeSR780@CAT@Mex-RS17 (+) (FeSR780: 2 μg/ml; CAT: 2 μg/ml; 0.5 W/cm^2^ and 0.5 min). LLC cells were incubated with 10 μM 2,7-dichlorodihydrofluorescein diacetate (H_2_DCFDA) for 30 min at 37 °C in the dark to detect ROS. The cells were then washed thrice and resuspended in 500 μl of fresh PBS. The samples were analyzed by CLSM and FCM.

### CRT, HMGB1, and ATP assays

LLC cells were seeded (2 × 10^5^ cells/well) in 6-well plates and treated with PBS, CAT@Mex-RS17, FeSR780@Mex-RS17 (+), or FeSR780@CAT@Mex-RS17 (+) for 24 h (FeSR780: 2 μg/ml; CAT: 2 μg/ml; 0.5 W/cm^2^ and 0.5 min). The concentrations of CRT and HMGB1 in the cell culture supernatants were measured using enzyme-linked immunosorbent assay (ELISA) kits (Beyotime), following the manufacturer’s instructions. Cells were stained with anti-CRT or anti-HMGB1 antibodies, followed by incubation with fluorescein-conjugated secondary FCM analysis after resuspending the cells in 200 μl of PBS. The same treatment groups were used for WB analysis and CLSM. The concentration of ATP in the cell culture supernatants was determined using Enhanced ATP Assay Kit (Beyotime).

### In vitro HIF-1α expression

LLC cells were seeded at a density of 2 × 10^3^/dish in confocal dishes for 24 h and then treated with PBS, Mex-RS17, CAT@Mex-RS17, FeSR780@Mex-RS17 (+), or FeSR780@CAT@Mex-RS17 (+) NPs for 24 h. To create hypoxic conditions, all cells were incubated in a hypoxic chamber using pH 6.5 media supplemented with 50 μM H_2_O_2_ (5% CO_2,_ 1% O_2_, and 94% N_2_) for 24 h [30]. LLC cells were stained with HIF-1α primary antibody followed by the corresponding secondary antibody, and fluorescence images were captured CLSM. The protein levels of HIF-1α in the treated cells were quantified by WB analysis, as previously described. HIF-1α and GAPDH were visualized on the films.

### In vitro activation of DCs

BMDCs were isolated from C57BL/6 mice, seeded (1 × 10^6^ cells/well) in 6-well plates, and incubated for 12 h to allow attachment. The serum-free DMEM was replaced with the culture medium 2 h prior to the co-culture experiment. To induce ICD, LLC cells were treated with various NPs (PBS, Mex-RS17, CAT@Mex-RS17, FeSR780@Mex-RS17 (+), or FeSR780@CAT@Mex-RS17 (+)) for 12 h (FeSR780: 2 μg/ml; CAT: 2 μg/ml; 0.5 W/cm^2^ and 0.5 min). Subsequently, the cells were collected and stained for 30 min with anti-CD80 and anti-CD86 monoclonal antibodies. After staining, all cells were washed thrice, resuspended in PBS, and analyzed by FCM. The levels of interferon-β, interleukin-6, tumor necrosis factor-α, and interleukin-12 p70 secreted from BMDCs were determined using ELISA kits.

### In vitro macrophage repolarization measured

The effect of various NPs on macrophage repolarization was assessed by FCM. RAW264.7 cells were seeded (1 × 10^6^ cells/well) in 6-well plates and treated with IL-4 (10 ng/ml) for 48 h to induce polarization into M2 macrophages. The cells were then exposed to the different NPs for 24 h. After treatment, cells were collected and stained with anti-F4/80, anti-CD86, and anti-CD206 antibodies for 30 min at 4 °C. Finally, the cells were resuspended in 200 μl of PBS and analyzed using FCM.

### Biodistribution of FeSR780@CAT@Mex-RS17

All animal procedures were conducted in strict compliance with guidelines approved by the Animal Care and Use Committee of Hebei Medical University. C57BL/6 mice (females, 6 to 7 weeks) received subcutaneous injections of LLC cells (1 × 10^6^ cells) on the right flank to establish LLC subcutaneous xenograft models. The mice were divided into 3 groups and injected via the tail vein with free ICG, ICG-FeSR780@CAT@Mex, or ICG-FeSR780@CAT@Mex-RS17 once the tumors reached approximately 400 mm^3^ in size. Whole-body live fluorescence imaging was performed at predetermined time points (1, 6, and 24 h) using an in vivo imaging system. After the final 24 h, the mice were euthanized, and the main organs, along with the tumor tissues, were collected for ex vivo fluorescence imaging.

### In vivo therapeutic efficacy

To establish the LLC subcutaneous model, 1 × 10^6^ LLC cells were subcutaneously injected into C57BL/6 mice on day 0, as described above. Tumor volume was measured every 3 d using an electronic caliper and calculated according to the following formula: *V* = length × width^2^/2. Changes in mouse weight were recorded simultaneously. Mice with palpable tumors (50 mm^3^) were randomly divided into 5 groups, with 6 mice per group. Each group received an intravenous injection of PBS, Mex-RS17, CAT@Mex-RS17, FeSR780@Mex-RS17, or FeSR780@CAT@Mex-RS17, respectively, at the following dosages: FeSR780 (4 mg/kg), CAT (4 mg/kg), and 1.5 W/cm^2^ for 6 min after 24 h postinjection. On the 18th day, the subcutaneous tumors were resected and weighed. Endpoint events were defined as follows: mortality, ulceration within the tumor tissue, tumor volume > 1,500 mm^3^, or weight loss > 15%.

### Lung orthotopic models and liver metastasis models

To assess the ability of FeSR780@CAT@Mex-RS17 NPs to inhibit tumor metastasis, an LLC subcutaneous xenograft mouse model was established on day −10, as described above. Tumors were resected on day 0 under anesthesia after receiving different treatments on day −1. LLC cells (3 × 10^6^) were injected intravenously to establish an orthotopic lung model. To establish the liver metastasis model, 5 × 10^5^ LLC cells were injected into the spleen of each mouse via laparotomy under anesthesia. The survival of mice was monitored daily. Ten LLC tumor-bearing mice were randomly divided into 2 groups: PBS and FeSR780@CAT@Mex-RS17 (+) (FeSR780: 4 mg/kg; CAT: 4 mg/kg; 1.5 W/cm^2^ and 6 min). Three intravenous injections were administered on days 0, 7, and 14. The mice were euthanized at the end of the experiment to collect the lungs and livers, which were then photographed and stained with hematoxylin and eosin (H&E).

### In vivo analysis of the immune response

After 18 d of treatment, mice were euthanized for immune cell analysis. Cytokine analysis was performed using mouse blood samples by ELISAs. Tumors and spleen were collected and subjected to immune analysis. Briefly, single-cell suspensions from tumors or spleens were prepared using a 70-μm filter after grinding. DC maturation was assessed by FCM after tumor cells were stained with anti-CD86 and anti-CD80 antibodies. Additionally, anti-F4/80, anti-CD86, and anti-CD206 antibodies were used to stain the cells generated from tumors and analyze their polarization by FCM. To assess M1/M2 macrophage polarization, cells generated from tumors were stained with anti-F4/80, anti-CD206, and anti-CD86 antibodies and analyzed using FCM. Splenocytes were obtained by centrifuging and washing the cells from the spleen after removing erythrocytes. Finally, the cells were stained with anti-CD3, anti-CD4, and anti-CD8 antibodies according to standard protocols for FCM analysis. To investigate long-term immune memory, C57BL/6 mice were treated as described above. On day 60, splenocytes were collected and stained with anti-CD3, anti-CD8, anti-CD44, and anti-CD62L antibodies for FCM analysis.

### In vivo safety analysis

The hemolytic activity of FeSR780@CAT@Mex-RS17 was measured in a 2% erythrocyte suspension mixed with different concentrations (10, 20, 50, 100, 200, and 500 μg/ml) of FeSR780@CAT@Mex-RS17. PBS was used as the negative control, and double-distilled water was used as the positive control. The absorbance of the supernatant was measured at a wavelength of 540 nm. Furthermore, to assess the long-term safety and biocompatibility of FeSR780@CAT@Mex-RS17, C57BL/6 mice (females, 6 to 7 weeks) were intravenously injected with FeSR780@CAT@Mex-RS17 NPs 4 times per month for 2 months (*n* = 5; FeSR780 4 mg/kg and CAT 4 mg/kg). Following the completion of the experiment, blood samples were collected for routine blood examination and serum biochemical index analysis to assess hepatic and renal toxicity. Additionally, the main organs were harvested and stained with H&E to observe tissue damage.

### Statistical analysis

Statistical comparisons between the 2 groups were conducted using the Student *t* test. The results are presented as mean ± standard error of the mean. Statistical significance was set at *P* < 0.05, and the data are indicated with * for *P* < 0.05, ** for *P* < 0.01, *** for *P* < 0.001, and **** for *P* < 0.0001. All statistical analyses were performed using the GraphPad Prism software.

## Results and Discussion

### Characterization of FeSR780@CAT@Mex-RS17

**Fig. 2.**
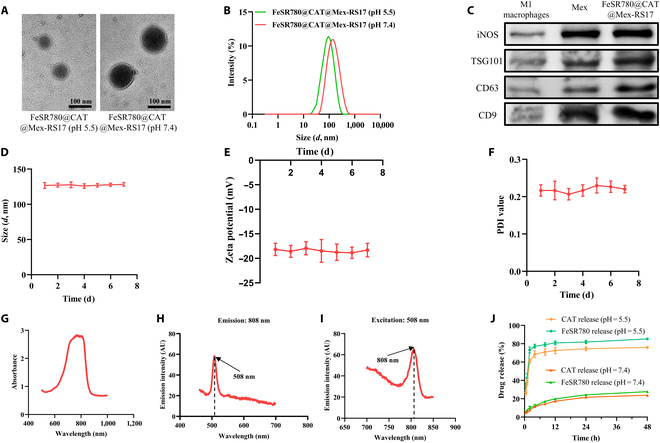
Characterization of FeSR780@CAT@Mex-RS17. Representative transmission electron microscopy (TEM) images (A) and size distribution (B) of FeSR780@CAT@Mex-RS17 at pH 5.5 or pH 7.4. (C) Protein expression in M1 macrophages, M1 exosomes, and SPI@hEL-RS17 NPs. The changes in size (D), zeta potential (E), and polydispersity index (PDI) value (F) of FeSR780@CAT@Mex-RS17 were evaluated after 7 d. (G) Ultraviolet–visible (UV–vis) spectrum of FeSR780. (H) Excitation spectrum of FeSR780 emitted at 808 nm, displaying a peak excitation at 508 nm. (I) The emission spectra of FeSR780 excited by 508-nm lasers, displaying a peak emission at 808 nm. (J) Catalase (CAT) release profile and FeSR780 release profile from FeSR780@CAT@Mex-RS17 at pH 5.5 or pH 7.4. CAT, catalase; iNOS, inducible nitric oxide synthase; TSG101, tumor susceptibility gene 101.

We prepared an acidity-activatable nanoplatform named FeSR780@CAT@Mex-RS17 NPs, as described in Fig. 1. TEM and DLS measurements were performed on FeSR780@CAT@Mex-RS17 NPs to determine their morphology and hydrated particle size. TEM images indicated that the spherical structure of the NPs had a diameter of ~130 nm at pH 7.4 (Fig. 2A). The FeSR780@CAT@Mex-RS17 NPs had a size of 128.3 ± 2.65 nm at pH 7.4, as measured by DLS. Notably, their size reduced to 93.33 ± 2.13 nm at pH 5.5, a condition that mimics the acidity [31] of the TME (Fig. 2B). This change in size suggests that FeSR780@CAT@Mex-RS17 NPs may undergo degradation under acidic conditions of the TME. The pH responsiveness of FeSR780@CAT@Mex-RS17 NPs was further confirmed by the observed size changes. WB analysis of FeSR780@CAT@Mex-RS17 NPs (Fig. 2C) revealed the presence of key exosomal marker proteins, including tumor susceptibility genes 101, CD63, and CD9 [32]. Moreover, inducible nitric oxide synthase, a protein typically associated with M1 macrophages, was detected, indicating that M1 macrophage function was preserved in the FeSR780@CAT@Mex-RS17 NPs. Furthermore, DLS, zeta potential, and polydispersity index measurements demonstrated that the FeSR780@CAT@Mex-RS17 NPs remained stably dispersed in FBS over 7 d (Fig. 2D to F). In its absorption spectrum, FeSR780 exhibited a maximum absorption wavelength of 808 nm (Fig. 2G). The emission and excitation spectra of FeSR780 are displayed in Fig. 2H and I, respectively. FeSR780@CAT@Mex-RS17 NPs were investigated in solutions of different pH values to evaluate the release behavior of FeSR780 and CAT in the TME (Fig. 2J). In contrast to the sluggish FeSR780 and CAT release behavior of FeSR780@CAT@Mex-RS17 NPs at pH 7.4, the release rates were faster in acidic solutions at pH 5.5. Specifically, within 4 h at pH 5.5, the release rates of FeSR780 and CAT were 77.3% ± 2.1% and 68.7% ± 3.7%, respectively, whereas less than 27.8% of both agents were released within 48 h at pH 7.4. These findings confirm that FeSR780@CAT@Mex-RS17 NPs are promising acid-responsive NPs for achieving efficient drug release.

As discussed previously, FeSR780@CAT@Mex-RS17 NPs have good stability in PBS. To mimic the physiological environment, the stability of the FeSR780@CAT@Mex-RS17 NPs within 7 d was measured in bovine serum albumin (BSA) solution. DLS, zeta potential, and polydispersity index measurements demonstrated that the FeSR780@CAT@Mex-RS17 NPs remained stably dispersed in BSA over 7 d (Fig. S1). The results show that FeSR780@CAT@Mex-RS17 has good stability in BSA, same as in PBS solution.

Considering that the acidity within the TME usually gradually decreases, we evaluated the release of the NPs across a broader range of pH levels to mimic real tumor conditions. As shown in Fig. S2, in an environment with pH 5.5, the cumulative release of FeSR780 reached approximately 83% at 48 h, which was approximately 1.3-fold, 1.4-fold, and 3.0-fold that of the cumulative release at pH 6.5, pH 6.8, and pH 7.4, respectively; the cumulative release of CAT reached approximately 76% at 48 h, which was approximately 1.3-fold, 1.5-fold, and 3.2-fold that of the cumulative release at pH 6.5, pH 6.8, and pH 7.4, respectively. The results confirmed that an acidic environment promoted the release of FeSR780 and CAT, and the release amount increased with the decrease in pH.

### Evaluation of cell uptake in vitro

**Fig. 3.**
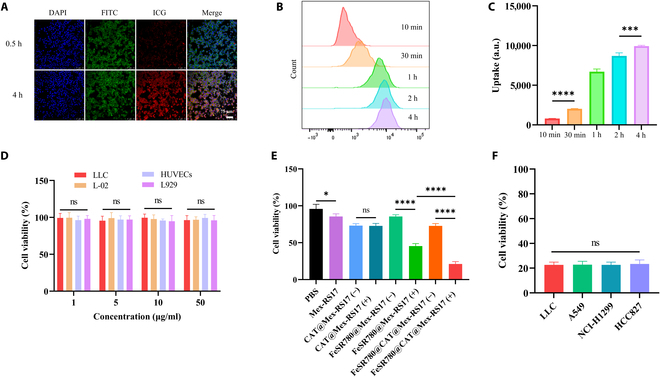
(A) Representative confocal laser scanning microscopy (CLSM), (B) flow cytometry (FCM) profiles, and (C) semiquantification of the cell uptake of FeSR780@CAT@Mex-RS17 by Lewis lung carcinoma (LLC) cells at different time points. Scale bar: 75 μm. (D) Viability of LLC, human umbilical vein endothelial cells (HUVECs), L-02, and L929 cells treated with blank Mex at different concentrations (1, 5, 10, and 50 μg/ml). (E) Viability of LLC cells treated with different treatments and (F) viability of LLC, A549, NCI-H1299, and HCC827 cells treated with FeSR780@CAT@Mex-RS17. (+) indicates irradiation with an 808-nm laser (1.5 W/cm^2^ and 6 min). DAPI, 4′,6-diamidino-2-phenylindole; FITC, fluorescein isothiocyanate; ICG, indocyanine green; ns, not significant; PBS, phosphate-buffered saline.

The in vitro cell uptake of FeSR780@CAT@Mex-RS17 NPs was analyzed using FCM and CLSM. Strong ICG fluorescent signals were detected in the CLSM images after incubation for 4 h (Fig. 3A). Cellular uptake was quantified by FCM. The uptake fold was 12.3 by dividing the fluorescence intensity of cells after incubation for 4 h by that after incubation for 10 min (Fig. 3B and C). These results demonstrate that FeSR780@CAT@Mex-RS17 exhibited excellent cellular uptake efficiency. The binding of tumor cells increased with increasing incubation time, illustrating that FeSR780@CAT@Mex-RS17 NPs were ingested by cells in a time-dependent manner.

### Antitumor activity in vitro

A CCK-8 assay was used to evaluate the cytotoxicity of the nanosystem. First, we found that blank Mex had negligible effects on the viability of a lung cancer cell line (LLC cells) and 3 normal cell lines (human umbilical vein endothelial cells, L-02 cells, and L929 cells) at concentrations ≤50 μg/ml, as determined by the CCK-8 assay, suggesting that our FeSR780@CAT@Mex-RS17 NPs were highly biocompatible (Fig. 3D). The CCK-8 assay demonstrated cell viabilities of 95.8% ± 6.3%, 85.6% ± 3.6%, 73.2% ± 2.6%, 72.8% ± 3.4%, 85.6% ± 2.3%, 45.4% ± 3.4%, 72.8% ± 3.1%, and 21.2% ± 3.2% after treatment with PBS, Mex-RS17, CAT@Mex-RS17 (−), CAT@Mex-RS17 (+), FeSR780@Mex-RS17 (−), FeSR780@Mex-RS17 (+), FeSR780@CAT@Mex-RS17 (−), and FeSR780@CAT@Mex-RS17 (+), respectively. Compared to the other groups, FeSR780@CAT@Mex-RS17 (+) importantly reduced LLC cell viability (*P* < 0.0001; Fig. 3E). Finally, the tumor-killing effect of FeSR780@CAT@Mex-RS17 (+) was evaluated in LLC, A549, NCI-H1299, and HCC827 cells. In all 4 cell lines, FeSR780@CAT@Mex-RS17 (+) demonstrated a marked tumor-killing ability (Fig. 3F).

### Ferroptosis in vitro

**Fig. 4.**
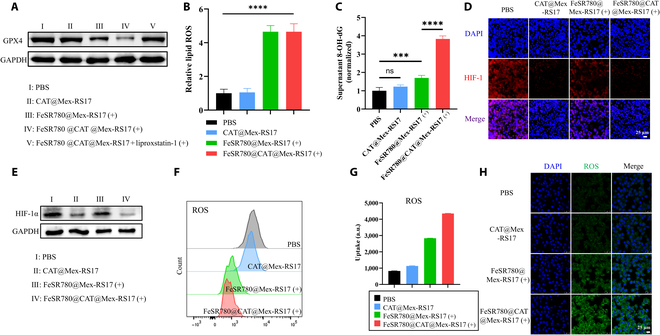
(A) Western blot (WB) assay of GPX4 expression in LLC cells incubated with different treatments. (B) FCM analysis of ferroptosis-induced intracellular accumulation of lipid peroxide in LLC tumor cells in vitro using the BODIPY-C11 fluorescent probe. (C) 8-Hydroxydeoxyguanosine (8-OH-dG) quantification in the cytosol of LLC cell supernatants following various treatments according to the manufacturer’s instructions (*n* = 4). CLSM (D) and WB (E) of HIF-1α expression in LLC cells in vitro under hypoxic conditions. Scale bar: 25 μm. (F) ROS generation in LLC cells after various treatments as determined by FCM and the corresponding quantification (G) of ROS generation and (H) ROS generation in LLC after various treatments using CLSM. Scale bar: 25 μm. GAPDH, glyceraldehyde-3-phosphate dehydrogenase.

The WB assay demonstrated that Fe^2+^ delivery by FeSR780@CAT@Mex-RS17 NPs importantly inhibited the expression of GPX4 in LLC cells (Fig. 4A), indicating that Fe^3+^ was efficiently released from the NPs, triggering the Fenton reaction. The ferroptosis inhibitor liproxstatin-1 reversed the down-regulation of GPX4. Collectively, these findings suggest that FeSR780@CAT@Mex-RS17 NPs can efficiently release Fe^3+^ for GSH depletion while simultaneously inhibiting GPX4 expression to induce ferroptosis. As ferroptosis disrupts cellular redox homeostasis, we measured the intracellular and lipid ROS levels in LLC cells treated with PBS (control), CAT@Mex-RS17, FeSR780@Mex-RS17 (+), or FeSR780@CAT@Mex-RS17 (+). FeSR780@CAT@Mex-RS17 NPs induced an ~4.7-fold higher level of ROS in intracellular lipids compared to the control group, indicating that FeSR780@CAT@Mex-RS17 NPs triggered GPX4-dependent lipid peroxidation in tumor cells (Fig. 4B). To elucidate the mechanism underlying mitochondrial DNA escape, we analyzed the 8-hydroxydeoxyguanosine content in the supernatants of LLC cells treated with various prepared formulations. Figure 4C shows that the FeSR780@CAT@Mex-RS17 NP group produced the highest levels of cytoplasmic 8-hydroxydeoxyguanosine.

### Expression of HIF-1α in vitro

Hypoxic conditions support HIF-1α stability, while normoxic conditions [33] lead to its degradation. Further confirmation of hypoxia relief was provided by the decrease in HIF-1α expression in cells treated with FeSR780@CAT@Mex-RS17 NPs (Fig. 4D and E). Therefore, FeSR780@CAT@Mex-RS17 NPs exhibit excellent capabilities for normalizing the pH, producing ROS, and alleviating hypoxia. Moreover, LLC cells treated with FeSR780@CAT@Mex-RS17 NPs reported the strongest fluorescence intensity after incubation with the ROS probe H_2_DCFDA (Fig. 4F and G), which were further quantified by CLSM (Fig. 4H). Consequently, the combination of PDT and ferroptosis is believed to synergistically enhance ICD effects.

As a result of the ROS generated by FeSR780@CAT@Mex-RS17 NPs, obvious oxidative damage can occur, which enhances the local immune response in the tumor. These findings demonstrate that FeSR780@CAT@Mex-RS17 NPs are equipped with highly efficient CAT nanozymes, which not only self-supply adequate oxygen but also deplete GSH and produce ROS, thereby disrupting the tumor’s reductive and immunosuppressive microenvironment defenses.

### ICD effect in vitro

**Fig. 5.**
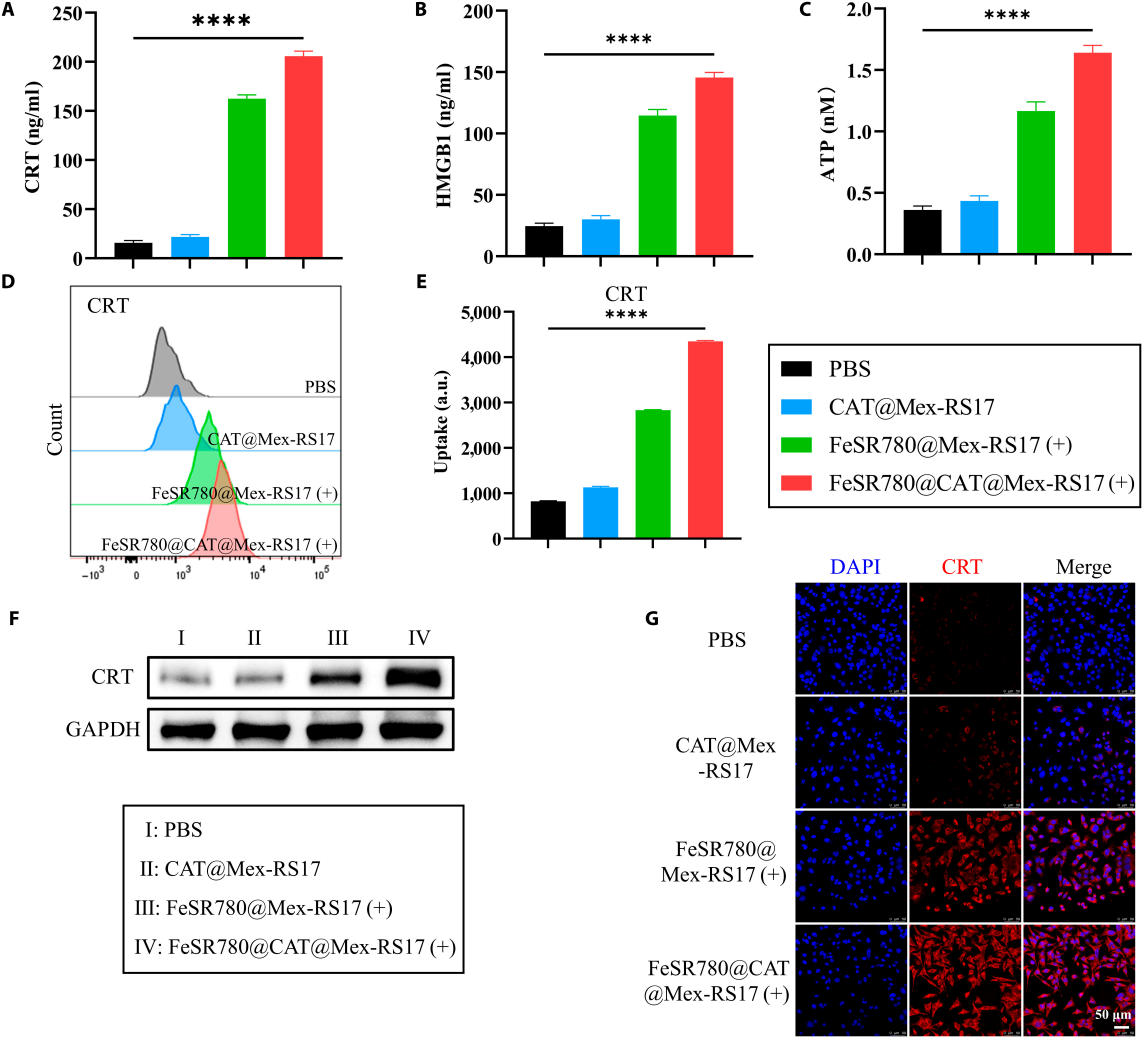
CRT (A), HMGB1 (B), and ATP (C) levels in LLC supernatants after different treatments. CRT exposure in LLC cells after various treatments: (D) FCM, (E) corresponding quantification, (F) WB analysis, and (G) CLSM of CRT exposure.

PDT has proven to be a useful ICD inducer [34,35]. PDT-induced ICD may lead to an increase in the exposure of DAMPs on the cell surface, signaling “eat me”. To validate this, we assessed the release of DAMPs from LLC cells treated with PBS, CAT@Mex-RS17 NPs, FeSR780@Mex-RS17 NPs, or FeSR780@CAT@Mex-RS17 NPs using CRT and HMGB1 ELISA Kits and an ATP assay kit. As demonstrated in Fig. 5A to C, the release of CRT, HMGB1, and ATP obviously increased following treatment with FeSR780@CAT@Mex-RS17 NPs. LLC cells treated with FeSR780@CAT@Mex-RS17 NPs exhibited a 13.0-fold increase in extracellular CRT levels, a 5.9-fold increase in HMGB1 release, and a 4.6-fold increase in ATP release compared to cells treated with PBS. Furthermore, LLC cells treated with FeSR780@CAT@Mex-RS17 NPs demonstrated an important increase in DAMP release compared to the CAT@Mex-RS17 NP group (CRT: 9.5-fold; HMGB1: 4.8-fold; ATP: 3.8-fold) and the FeSR780@Mex-RS17 NP group (CRT: 1.3-fold; HMGB1: 1.3-fold; ATP: 1.4-fold). To further explore the mechanisms involved, we analyzed the protein expression levels and fluorescence of CRT using FCM, WB, and CLSM. These results are consistent with previous findings (Fig. 5D to G). Overall, these findings demonstrated that FeSR780@CAT@Mex-RS17 NPs efficiently induced ICD in LLC cells.

### Immune response in vitro

**Fig. 6.**
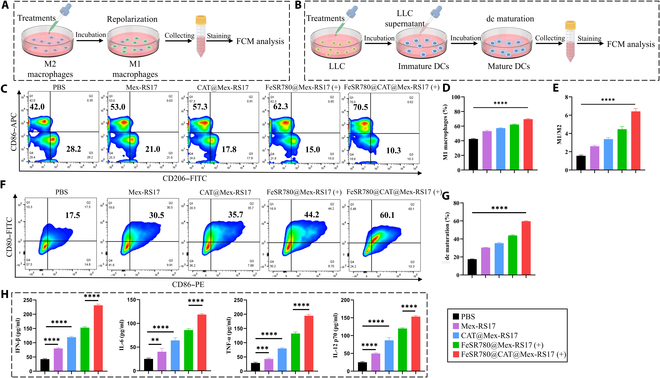
(A) Illustration of macrophage repolarization by different treatments. (B) Illustration of DC maturation under different treatments. (C) Representative FCM and quantitative analysis (D and E) of macrophage repolarization in response to various treatments. (F) Analysis of DC maturation following different treatments by FCM and quantitative analysis (G); (H) the cytokine levels of interferon-β (IFN-β), interleukin-6 (IL-6), tumor necrosis factor-α (TNF-α), and interleukin-12 (IL-12) p70 in DC supernatants were measured using enzyme-linked immunosorbent assay (ELISA) (*n* = 4).

The immunosuppressive TME is characterized by infiltration of M2 macrophages into “cold” tumors [15]. Anti-inflammatory and immunosuppressive M2-like phenotypes are commonly found in tumor-associated macrophages (TAMs) within the TME, contributing to immune evasion and tumor progression [36]. In an immunosuppressive TME, the transition of TAMs from the protumor (M2) phenotype to the antitumor (M1) phenotype is of great importance [37]. Oxidative stress has also been linked to increased ROS production, as shown in previous studies [38]. Consequently, inspired by the outstanding ability of FeSR780@CAT@Mex-RS17 NPs to generate ROS and induce the up-regulation of ICD markers, immune activation experiments were performed using LLC cells to investigate whether FeSR780@CAT@Mex-RS17 NPs could effectively activate the antitumor immune system. RAW264.7 macrophages were treated with IL-4 to establish M2 macrophages and incubated with FeSR780@CAT@Mex-RS17 NPs, effectively repolarizing M2 macrophages to M1 macrophages, which confirmed the polarization of M2 TAMs induced by FeSR780@CAT@Mex-RS17 NPs. RAW264.7 cells were then exposed to PBS, Mex-RS17 NPs, CAT@Mex-RS17 NPs, FeSR780@Mex-RS17 NPs, or FeSR780@CAT@Mex-RS17 NPs, and the proportions of M2 and M1 macrophages were analyzed using FCM (Fig. 6A). An illustration of DC maturation after different treatments is presented in Fig. 6B. As demonstrated in Fig. 6C to E, Mex-RS17 NPs, CAT@Mex-RS17 NPs, FeSR780@Mex-RS17 NPs, and FeSR780@CAT@Mex-RS17 NPs all obviously promoted the repolarization of RAW264.7 cells to an M1-like phenotype, as indicated by the increased expression of CD86, with FeSR780@CAT@Mex-RS17 NPs demonstrating the strongest effect among all groups. Compared with the control, the proportion of M2 macrophages in the FeSR780@Mex-RS17 NP and FeSR780@CAT@Mex-RS17 NP groups decreased obviously from 27.4% to 14.0% and 10.9%, respectively, while the proportion of M1 macrophages increased from 42.5% to 62.2% and 69.6%, respectively. Furthermore, we explored whether FeSR780@CAT@Mex-RS17 NPs could promote DC maturation in vitro. DCs serve as specialized antigen-presenting cells that present tumor antigens to T cells. For the DC stimulation experiment, LLC cells were pretreated with PBS, Mex-RS17 NPs, CAT@Mex-RS17 NPs, FeSR780@Mex-RS17 NPs, or FeSR780@CAT@Mex-RS17 NPs, and supernatants were collected and co-cultured with BMDCs. We then evaluated the expression of costimulatory molecules CD86 and CD80 in BMDCs using FCM. As displayed in Fig. 6F and G, LLC cells treated with FeSR780@CAT@Mex-RS17 increased the expression of CD80 and CD86 in BMDCs, and the proportion of mature DCs increased from 17.5% to 59.7% compared with that in the control, indicating that FeSR780@CAT@Mex-RS17 NPs promoted the maturation and activation of DCs. An important increase in cytokine (interferon-β, interleukin-6, tumor necrosis factor-α, and interleukin-12 p70) levels was observed in the culture medium following treatment with FeSR780@CAT@Mex-RS17 NPs (+) (*P* < 0.0001; Fig. 6H). According to these results, FeSR780@CAT@Mex-RS17 NP-mediated immunotherapy enhances antitumor responses by activating DCs.

### Biodistribution of FeSR780@CAT@Mex-RS17 in vivo

To further investigate the tumor-specific targeting of the nanovesicles, LLC tumor-bearing mice were intravenously injected with ICG-labeled FeSR780, ICG-labeled Mex, or ICG-labeled Mex-RS17 NPs and imaged at various time points (Fig. S3). The maximum fluorescence signal was observed at the tumors in all 3 groups at 24 h. Mice injected with ICG-Mex-RS17 NPs exhibited stronger fluorescence compared with the ICG-Mex group, indicating that RS17 modification obviously improves the targeting ability of Mex. The ICG-FeSR780 group exhibited lower fluorescence than the ICG-Mex group, indicating that the Mex derived from macrophages have good tumor-targeting ability. Taken together, the enhanced permeability and retention effect of NPs, the tumor-targeting ability of Mex, and RS17-labeling modification obviously improved tumor-specific targeting ability and overcome biological barriers in vivo.

**Fig. 7.**
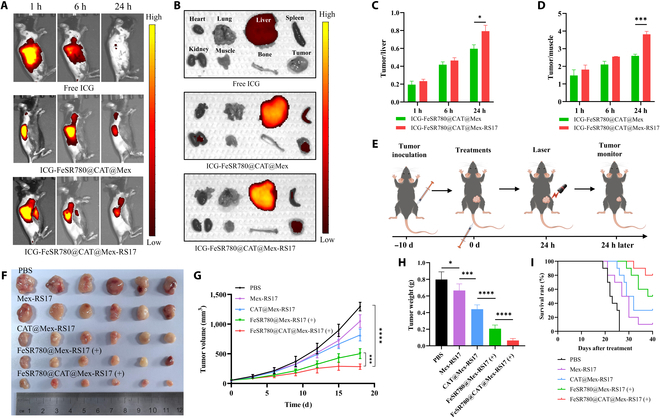
Biodistribution and antitumor efficacy of FeSR780@CAT@Mex-RS17 (+) in vivo. (A) In vivo fluorescence images of LLC tumor-bearing mice at predetermined time points. (B) Ex vivo fluorescence images of major organs and tumors collected 24 h postinjection. (C and D) Semiquantitative analysis of fluorescence intensity in the images. (E) Schematic representation of the mouse treatment regimen. (F) Representative tumor photographs of LLC tumor-bearing mice on day 18 after various treatments (*n* = 6). Tumor volume (G), tumor weight (H), and survival curves (I) of LLC tumor-bearing mice that received different treatments.

Subsequently, we studied the distribution of FeSR780@CAT@Mex-RS17 by in vivo imaging of LLC tumor-bearing mice after intravenous injection. As presented in Fig. 7A, ICG-FeSR780@CAT@Mex-RS17 and ICG-FeSR780@CAT@Mex exhibited maximal tumor accumulation 24 h after intravenous injection compared with free ICG. The fluorescence intensities of ICG-FeSR780@CAT@Mex-RS17 and ICG-FeSR780@Mex-RS17 were 5.1-fold and 2.2-fold higher, respectively, than that of free ICG in tumor tissues (Fig. S4a). In solution, negatively charged NPs (such as FeSR780@CAT@Mex-RS17) are considered stable and demonstrate prolonged blood circulation time and high tumor uptake [39,40]. As a result of the enhanced permeability and retention effect of the Mex delivery platform, along with the RS17 peptide and CD47 interaction specifically targeting tumor cells, FeSR780@CAT@Mex-RS17 NPs demonstrated prolonged intratumoral retention and increased accumulation at tumor sites. Furthermore, ex vivo imaging (Fig. 7B) of tumors from the ICG-FeSR780@CAT@Mex-RS17 NP group demonstrated an obviously enhanced ICG signal 24 h postinjection, indicating that the NPs were highly effective in targeting and accumulating in the tumors. Semiquantitative data based on ex vivo images (Fig. 7C and D and Fig. S4b) further confirmed that the tumor/liver and tumor/muscle ratios at 24 h were higher in the ICG-FeSR780@CAT@Mex-RS17 NP group (tumor/liver: 0.79 ± 0.07; tumor/muscle: 3.82 ± 0.17) than in the ICG-FeSR780@CAT@Mex NP group (tumor/liver: 0.60 ± 0.04; tumor/muscle: 2.60 ± 0.09), indicating that tumor targeting was obviously improved by the conjugation of the RS17 peptide. Furthermore, fluorescence was observed in the liver and spleen tissues, likely because of their role in the clearance of FeSR780@CAT@Mex-RS17 NPs.

### Antitumor efficacy of FeSR780@CAT@Mex-RS17 NPs (+) in vivo

To evaluate whether FeSR780@CAT@Mex-RS17 could suppress tumor growth in immunocompetent mice, we investigated the therapeutic effect of FeSR780@CAT@Mex-RS17 in a subcutaneous xenograft tumor model using LLC cells. Various NPs were injected intravenously into the tumors when they reached ~50 mm^3^ in size (Fig. 7E). After 24-h postinjection of FeSR780@CAT@Mex-RS17 NPs, near-infrared laser irradiation was applied (808 nm at 1.5 W/cm^2^ for 6 min; FeSR780, 4 mg/kg; and CAT, 4 mg/kg). Tumor growth was monitored every 3 d following treatment. At the end of the 18-d testing period, mice were sacrificed for ex vivo analysis. As indicated in Fig. 7F, all treatment groups exhibited varying degrees of tumor suppression compared to the PBS group. Notably, the FeSR780@CAT@Mex-RS17 NP (+) treatment resulted in tumor growth inhibition, with an inhibition rate of 77.1% (Fig. 7G). Following this, all tumors were collected and weighed, and the tumor growth inhibition rate (TGI) for each mouse was calculated accordingly [41]. As presented in Fig. 7H, FeSR780@CAT@Mex-RS17 NPs (+) demonstrated a remarkably high tumor growth inhibition rate (TGI: 117.2%) compared to the PBS control. TGI was calculated using the following formula: TGI = (1 − average tumor weight of the treatment group/average tumor weight of the control group) × 100%. Following treatment, the survival of mice was monitored for 40 d (Fig. 7I). The group treated with FeSR780@CAT@Mex-RS17 NPs (+) had the highest survival rate (80%).

Considering the diversity of clinical medication regimens, we further explored the effects of different medication regimens. The drug administration scheme is described in Fig. S5a to c. The LLC tumor-bearing mice were equally divided into 4 groups: single-intravenous-injection group, multiple-intravenous-injection group, single-intratumor-injection group, and untreated control group. As shown in Fig. S5d, all treatment groups exhibited marked degrees of tumor suppression compared to the untreated control group. In particular, FeSR780@CAT@Mex-RS17 NPs (+) by single-intratumor-injection treatment resulted in tumor growth inhibition, with an inhibition rate of 78.8%. In addition, single intravenous injection was as almost effective as multiple intravenous injections in maintaining reduction tumor growth, with inhibition rates of 73.0% and 75.1%, respectively. Finally, the treatment effect of FeSR780@CAT@Mex-RS17 NPs (+) was evaluated on different tumor types. As shown in Fig. S5e and f, FeSR780@CAT@Mex-RS17 NPs (+) by single-intravenous-injection treatment resulted in tumor growth inhibition in liver cancer (Hepa1-6 cell) and breast cancer (4T1 cell) models. In summary, our designed nanoparticles provided a platform to treat different types of cancers extensively through various treatment schemes, which may contribute to clinical application.

**Fig. 8.**
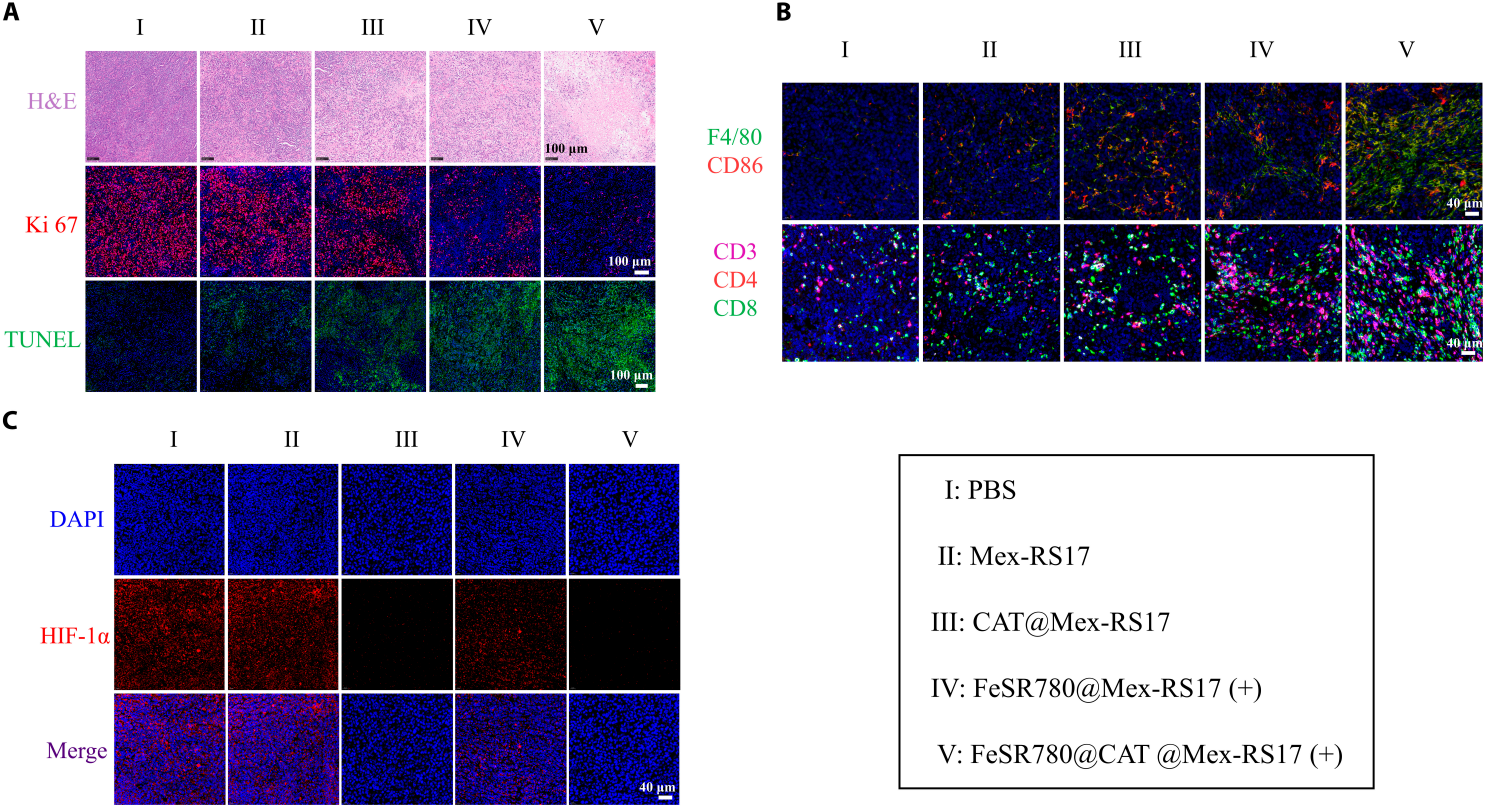
(A) Hematoxylin and eosin (H&E), Ki 67, and terminal deoxynucleotidyl transferase dUTP nick end labeling (TUNEL) stainings of LLC tumor tissues after treatment. Scale bar: 100 μm. (B) Immunofluorescence images displaying M1 macrophages and CTL infiltration into the tumor tissues. Scale bar: 40 μm. (C) HIF-1α expression in LLC tumor tissues after various treatments. Scale bar: 40 μm.

To further assess in vivo antitumor efficacy, H&E, Ki 67, and terminal deoxynucleotidyl transferase dUTP nick end labeling (TUNEL) staining were performed. The results indicated that the FeSR780@CAT@Mex-RS17 group had the lowest Ki 67 proliferation index and highest TUNEL levels among the 5 groups (Fig. 8A). Furthermore, tumor sections from the FeSR780@CAT@Mex-RS17 NP group revealed a marked increase in the infiltration and activation of M1 macrophages and CTLs in the TME (Fig. 8B). This highlights the role of FeSR780@CAT@Mex-RS17 in promoting macrophage M1 polarization and enhancing CD8^+^ T cell infiltration. Furthermore, HIF-1α (green) and DAPI (blue) staining revealed a marked reduction in HIF-1α levels in the FeSR780@CAT@Mex-RS17 group (Fig. 8C), indicating that the treatment successfully alleviated tumor hypoxia.

Hypoxia is known to be a key factor in the TME [42], which not only promotes tumor growth, advancement, and spread but also causes resistance to chemotherapy in terms of pharmacokinetics and pharmacodynamics [43]. HIF-1α is widely recognized as a crucial marker of tumor hypoxia, influencing tumor cell growth, blood vessel formation, anaerobic glycolysis, and resistance to hypoxia-related treatments. In addition, numerous indicators are also strongly associated with tumor hypoxia. Carbonic anhydrase IX (CAIX) is found to be overexpressed across various cancer types and has been promoted as a promising biomarker for targeting hypoxia in the TME [44]. Under low oxygen levels, CAIX supports the survival of cancer cells and is connected to an increased risk of metastasis. Activating transcription factor 4 (ATF4), a transcription factor induced by stress, is frequently triggered in hypoxic conditions [45]. ATF4 regulates the expression of various adaptive genes that enable cells to survive under conditions of low oxygen or limited amino acids. Immunosuppression in the TME is influenced by disordered metabolic states, which are identified by hypoxia and increased levels of metabolites like lactate [46]. Lactate production typically rises in tumors under low-oxygen conditions. The enzyme lactate dehydrogenase A (LDHA) plays a crucial role in glycolysis by catalyzing the transformation of pyruvate to l-lactic acid. To further investigate the capability of FeSR780@CAT@Mex-RS17 NPs to relieve tumor hypoxia, we detected the expression of CAIX, ATF4, and LDHA in vivo under hypoxic conditions by WB analysis. As shown in Fig. S6, FeSR780@CAT@Mex-RS17 NPs treatment led to remarkable down-regulation of CAIX, ATF4, and LDHA, indicating that tumor hypoxia was effectively relieved.

### Immune response in vivo

**Fig. 9.**
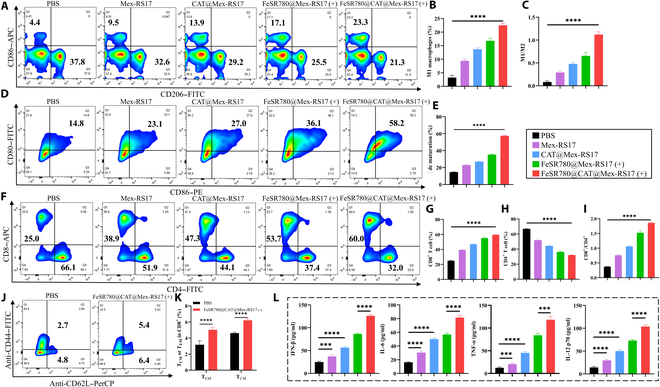
In vivo immune response induced by FeSR780@CAT@Mex-RS17 (+). (A) Representative FCM analysis of macrophage repolarization in tumor tissues and quantitative analysis (B and C) of macrophage repolarization (*n* = 4). (D) Representative FCM analysis of DC maturation in tumor tissues and quantitative analysis (E) of DC maturation (*n* = 4). (F) Representative FCM analysis of CTLs in tumors and quantitative analysis (G to I) of CTLs in tumor tissues (*n* = 4). (J) Representative FCM analysis of memory T cells in the spleen, displaying the proportions of CD8^+^CD44^+^CD62L^+^ (T_CM_) and CD8^+^CD44^+^CD62L^−^ (T_EM_) cells and quantitative analysis (K) of T_CM_ and TEM proportions (*n* = 4) and (L) serum cytokine levels (IFN-β, IL-6, TNF-α, and IL-12 p70) determined by ELISA following different treatments (*n* = 4).

Tumor acidity, lactate accumulation, and hypoxia have been reported to promote anergy in effector T lymphocytes and lead to the accumulation of suppressive immune cells, resulting in an immunosuppressive TME [47]. We assessed the activation of antitumor immunity using FeSR780@CAT@Mex-RS17 NPs. The percentages of M1 cells (F4/80^+^CD86^+^) and DCs (CD86^+^CD80^+^) were higher in the FeSR780@CAT@Mex-RS17 group (DCs: 57.4% ± 0.7%; M1 cells: 22.5% ± 0.8%) compared with that in the control group (DCs: 14.7% ± 0.2%; M1 cells: 3.2% ± 0.8%), indicating innate immune activation (Fig. 9A to E). FCM revealed that treatment with FeSR780@CAT@Mex-RS17 was most effective in increasing the number of tumor-infiltrating CD3^+^CD8^+^ T cells (Fig. 9F). This treatment not only increased the infiltration of CD3^+^CD8^+^ T cells into the tumor but also decreased the frequency of CD3^+^CD4^+^ T cells within the tumor (Fig. 9G and H). Consequently, the intratumoral ratio of CD8^+^/CD4^+^ T cells was up-regulated and ~5.0-fold higher than that in the control group (Fig. 9I). To explore the involvement of FeSR780@CAT@Mex-RS17 NPs in the long-term memory immune response, we collected splenocytes and analyzed the populations of immune memory cells (Fig. 9J). The percentage of effector memory T cells (TEM, CD8^+^CD44^+^CD62L^−^) in the FeSR780@CAT@Mex-RS17 group demonstrated a marked increase (~1.6-fold) compared to that in the control group(Fig. 9K). Moreover, elevated serum cytokine levels measured by ELISA indicated a strong protective immune response induced by FeSR780@CAT@Mex-RS17 (+) (Fig. 9L). Collectively, these findings demonstrated that FeSR780@CAT@Mex-RS17 NPs could efficiently induce antigen-specific antitumor immunity in tumors, suggesting their potential for broader applications in cancer treatment in the future.

### Antitumor effect in lung orthotopic and liver metastasis models

**Fig. 10.**
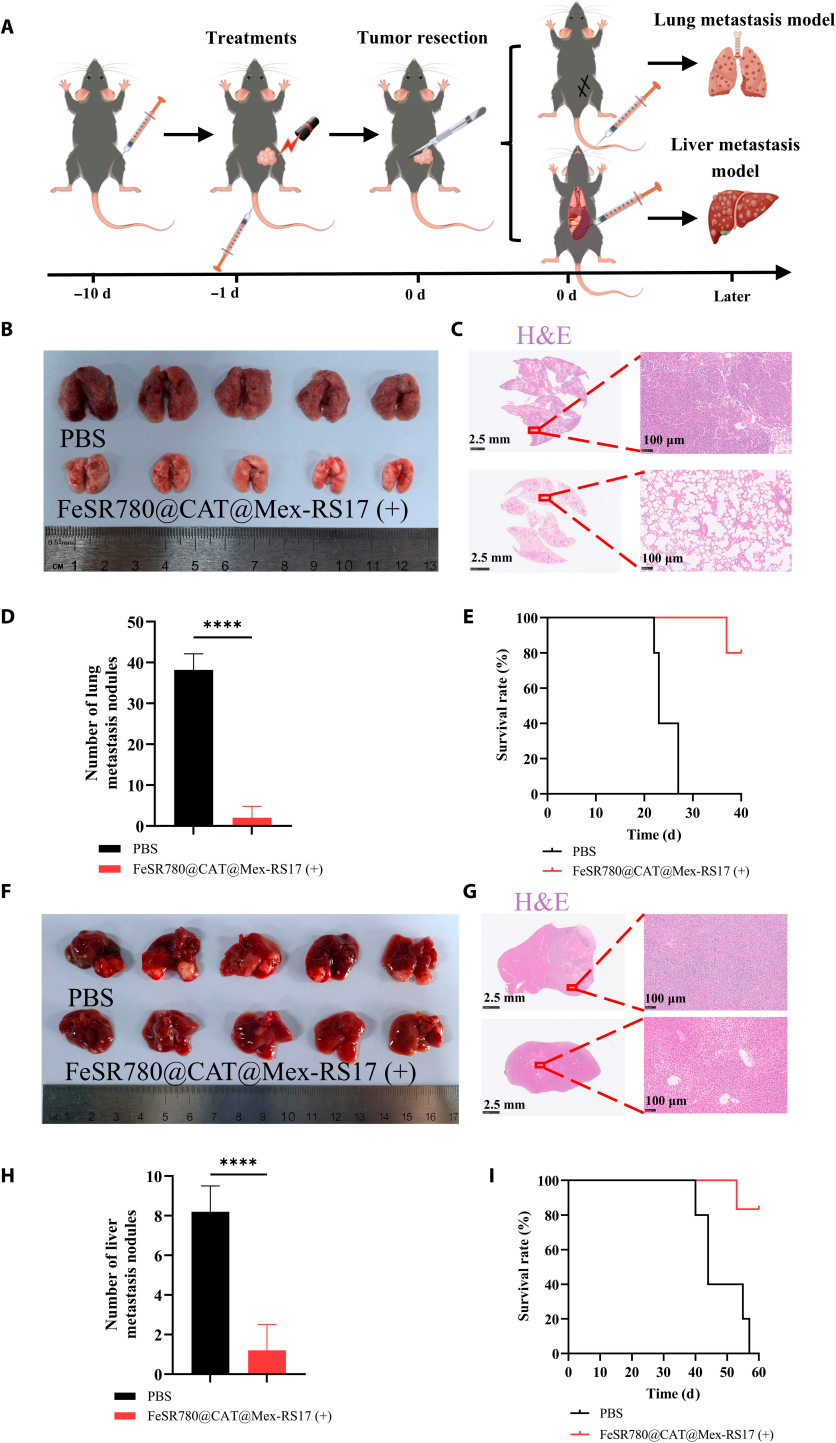
Antitumor efficacy in orthotopic lung and liver metastasis mouse models. (A) Schematic diagram illustrating the treatment schedule. Representative images of excised lung tissues (B) and the corresponding H&E-stained lung sections (C) on day 40. Scale bars: 2.5 mm (whole tissue) and 100 μm (locally enlarged views). (D) Quantification of the lung nodules (*n* = 5). (E) Survival curves of orthotopic LLC lung mice following various treatments (*n* = 5). Representative images of excised liver tissues (F) and the corresponding H&E-stained liver sections (G) on day 60. Scale bar: 2.5 mm (whole tissue) and 100 μm (locally enlarged views). (H) Quantification of liver metastasis nodules (*n* = 5) and (I) survival curves of LLC liver metastasis mice following various treatments (*n* = 5).

A major challenge in cancer therapy is combating metastasis, which accounts for over 90% of all cancer-related deaths [48]. Combination therapies for cancer treatment are generally more effective than monotherapy in maintaining tumor suppression [49]. Our research aimed to identify effective therapies that inhibit the growth of primary tumors and prevent metastasis by utilizing high-efficiency nanoplatforms that activate cytotoxicity, target tumors, and inhibit metastasis. Given the excellent immunotherapy potential of FeSR780@CAT@Mex-RS17 in the subcutaneous tumor model, we assessed its efficacy in orthotopic lung and liver metastasis models (Fig. 10A). In the LLC lung orthotopic model, the lung tissues of the 2 groups of C57BL/6 mice were harvested and lung nodules were quantified on day 40. Representative photographs of the lungs are presented in Fig. 10B. The lungs of mice were stained with H&E (Fig. 10C). Clear differences were observed between the FeSR780@CAT@Mex-RS17 (+) and PBS control groups in nodule detection (Fig. 10D). Moreover, treatment with FeSR780@CAT@Mex-RS17 (+) increased the survival rate from 0% in the control group to 80% in the tumor-bearing mice (Fig. 10E). In the LLC liver metastasis model, livers were collected on day 60 and photographed (Fig. 10F). H&E staining of liver sections (Fig. 10G) revealed an obvious reduction in the number of metastatic nodules in the FeSR780@CAT@Mex-RS17 (+) group (8.2 ± 1.3) compared to that in the control group (1.2 ± 1.3) (Fig. 10H). In the 60-d survival experiments, 80% of the mice in the FeSR780@CAT@Mex-RS17 (+) group survived, whereas none of the mice in the control group survived (Fig. 10I). Based on these findings, FeSR780@CAT@Mex-RS17 (+) mice demonstrated a marked reduction in LLC tumor metastases by inducing robust immune responses within the system.

### Safety evaluation of FeSR780@CAT@Mex-RS17 NPs in vivo

**Fig. 11.**
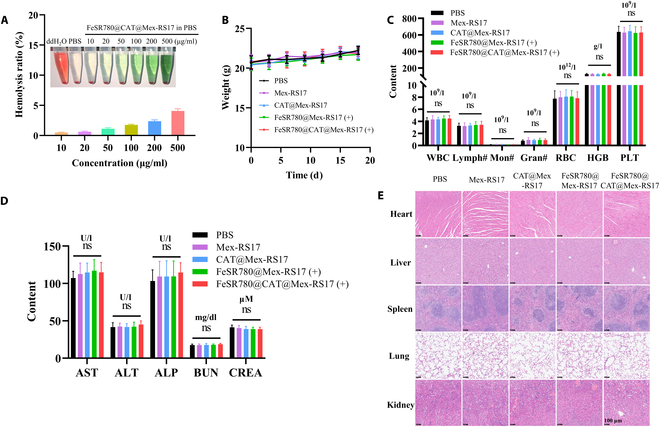
In vivo safety evaluation of FeSR780@CAT@Mex-RS17 NPs. (A) Images of erythrocyte suspensions incubated with various concentrations of FeSR780@CAT@Mex-RS17 NPs. Hemolysis ratios at different FeSR780@CAT@Mex-RS17 concentrations (*n* = 4), with PBS as the negative control and double-distilled water as the positive control. (B) Body weight changes in mice over 18 d. (C) Analysis of major routine blood parameters. (D) Measurement of liver and kidney function markers in blood biochemistry and (E) H&E-stained images of major organ slices. Scale bar: 100 μm. ddH_2_O, double-distilled water; WBC, white blood cells; Lymph#, lymphocyte count; Mon#, monocyte count; Gran#, granulocyte count; RBC, red blood cells; HGB, hemoglobin; PLT, platelet; AST; aspartate aminotransferase; ALT, alanine aminotransferase; ALP, alkaline phosphatase; BUN, blood urea nitrogen; CREA, creatinine.

Finally, the safety of FeSR780@CAT@Mex-RS17 NPs in vivo was assessed by conducting a hemolysis test, monitoring the body weight of the mice, analyzing routine blood and biochemical markers, and performing H&E staining of tumor sections. The hemolysis test, conducted with different concentrations of FeSR780@CAT@Mex-RS17, indicated that it did not cause obvious hemolysis at various concentrations (Fig. 11A). Even at the highest tested concentration of FeSR780@CAT@Mex-RS17 NPs (500 μg/ml), negligible hemolysis of less than 5% was observed, which met the specific compliance requirement of the 5% upper limit [50]. No substantial adverse effects or weight changes were observed in mice during the treatment period (Fig. 11B). Mice in all the treatment groups exhibited normal routine blood test parameters (Fig. 11C). Furthermore, after treatment with FeSR780@CAT@Mex-RS17, the levels of aspartate aminotransferase, alanine aminotransferase, blood urea nitrogen, and serum creatinine were nonsignificantly increased compared with those in the control group, suggesting that FeSR780@CAT@Mex-RS17 did not cause injury to liver and kidney tissues (Fig. 11D). Finally, H&E staining of the heart, liver, spleen, lung, and kidney tissues confirmed that there were no toxic side effects following treatment with FeSR780@CAT@Mex-RS17 (Fig. 11E). Overall, these findings indicate that FeSR780@CAT@Mex-RS17 has an excellent safety profile in vivo, making it a promising candidate for future clinical antitumor immunotherapy.

To further assess the long-term toxicity of the FeSR780@CAT@Mex-RS17 in vivo, FeSR780@CAT@Mex-RS17 was systematically investigated after intravenous injection into normal C57BL/6 mice (females, 6 to 7 weeks). Neither death nor obvious toxicity was observed at a specific time. Mice were sacrificed after injection on 30 d, and major organs such as livers, kidneys, and spleens were stained with H&E for histological analysis (Fig. S7a). No severe hepatic or renal toxicities were observed. No obvious abnormalities were detected in the blood routine examination and liver and kidney functions (Fig. S7b and c). Notably, FeSR780@CAT@Mex-RS17 is mainly metabolized by the liver and spleen, which observed negligible damage. Collectively, FeSR780@CAT@Mex-RS17 did not show abnormal activation or inhibition of immune cells.

This study has some limitations. First, it is unfeasible to use PDT for internal tumors, owing to the limited penetration of light. Moreover, testing this strategy in larger animal models requires extensive dose optimization of the designed NPs, which remains unclear and warrants further investigation. There is still a need to improve drug delivery efficiency and enhance the performance of PDT.

## Conclusion

In this study, we successfully developed intracellular-acidity-activatable FeSR780@CAT@Mex-RS17 for eliciting ferroptosis and promoting an antitumor immune response. The NPs were derived from the exosomes of M1 macrophages, decorated with the RS17 peptide, and coassembled with the photosensitizer SR780, Fe^3+^, and the antioxidant enzyme CAT. The NPs effectively targeted tumor cells and remodeled TAM phenotypes. Upon reaching the tumor site, the FeSR780@CAT@Mex-RS17 NPs undergo hydrolysis in response to the acidic TME. An enhanced antitumor effect is achieved through the combination of Fe^2+^-induced ferroptosis, SR780-induced PDT, and triggered ICD effects, along with close synergy among each component. In vivo studies demonstrated that FeSR780@CAT@Mex-RS17 NPs exhibited low systemic toxicity in a lung cancer mouse model, induced DC maturation, and enhanced tumor infiltration by CD8^+^ T cells, resulting in a better antitumor effect. Moreover, mice injected with FeSR780@CAT@Mex-RS17 NPs exhibited strong immunological memory, as evidenced by a substantially greater proportion of effector memory T cells. Consequently, the FeSR780@CAT@Mex-RS17 NPs presented a highly potent photodynamic immunotherapy approach that not only eliminates primary tumors but also provides abscopal effects to prevent liver metastasis and triggers the establishment of immunological memory. With further drug development for clinical applications, this strategy holds great potential for widespread use in cancer treatments.

## Data Availability

The analyzed datasets generated during this study are available from the corresponding authors on reasonable request.

## Supplementary Materials

Supplementary 1 Supplementary Text Table S1 Figs. S1 to S7 Western blot bands
