# Supplementary material for: pH-Activated Nanoplatform Derived from M1 Macrophages’ Exosomes for Photodynamic and Ferroptosis Synergistic Therapy to Augment Cancer Immunotherapy
Source: Biomater Res. 2025 Mar 6;29:0153. doi: 10.34133/bmr.0153 (PMC11883086; doi:10.34133/bmr.0153)
Supplement: Supplementary 1 — Supplementary Text Table S1 Figs. S1 to S7 Western blot bands [file bmr.0153.f1.zip › 4.supporting.docx]

Table S1. Antibodies used in the study.

| Antibodies | Source | Catalog |
| --- | --- | --- |
| iNOS | Abclonal | A3774 |
| TSG101 | Abclonal | A2216 |
| CD63 | Abclonal | A5271 |
| CD9 | Abclonal | A19027 |
| GPX4 | Abclonal | A1933 |
| HIF-1α | Abclonal | A22041 |
| Anti-mouse CRT | Abclonal | A1066 |
| Anti-mouse GAPDH | Abclonal | AC001 |
| HRP Goat Anti-Rabbit IgG(H+L) | Abclonal | AS014 |
| Abflo 594-conjugated Goat Anti-Rabbit IgG(H+L) | Abclonal | AS039 |
| FITC Goat Anti-Mouse IgG (H+L) | Abclonal | AS001 |
| APC Anti-Mouse CD86 | Elabscience | E-AB-F0994UE |
| FITC Anti-Mouse CD206 | Elabscience | E-AB-F1135C |
| PE Anti-Mouse CD86 | Elabscience | E-AB-F0994UD |
| FITC Anti-Mouse CD80 | Elabscience | E-AB-F0992UC |
| PE Anti-Mouse F4/80 | Elabscience | E-AB-F0995D |
| PerCP Anti-Mouse CD62L | Elabscience | E-AB-F1011J |
| FITC Anti-Mouse CD44 | Elabscience | E-AB-F1100C |
| PE anti-mouse CD3 | BioLegend | 100205 |
| FITC anti-mouse CD4 | BioLegend | 100405 |
| APC anti-mouse CD8 | BioLegend | 100711 |



Fig. S1. The changes in size (a), zeta potential (b), and PDI value (c) of FeSR780@CAT@Mex-RS17 in BSA were evaluated after seven days.



Fig. S2. The release of FeSR780 (a) and CAT (b) from FeSR780@CAT@Mex-RS17 at different pH values.

**

**

Fig. S3. *In vivo* fluorescence images of LLC tumor-bearing mice after different treatments at predetermined time points.





Fig. S4. a. Semiquantitative analysis of fluorescence intensity in tumor tissues. b. Semiquantitative analysis of fluorescence images in tumor, liver, and muscle tissues.





Fig. S5. (a-c) Schematic representation of the mice treatment regimen. (d) Tumor volume of LLC tumor-bearing mice that received different treatments. Tumor volumes of Hepa1-6 tumor-bearing mice (e) and 4T1 tumor-bearing mice (f) that received different treatments.

**

**

Fig. S6. WB of hypoxia-related proteins expression in LLC cells *in vivo* under the hypoxic condition.





Fig. S7. (a) H&E-stained images of major organ slices. Scale bar: 100 μm. (b) Analysis of major routine blood parameters. (c) Measurement of liver and kidney function markers in blood biochemistry.

**Materials**

DMEM medium, RPMI-1640 medium, phosphate buffer (PBS), fetal bovine serum (FBS), trypsin-EDTA, dimethyl-sulfoxide (DMSO), 100 U/mL penicillin, 100 mg/mL streptomycin, CCK8 kit, BCA kit, mouse TNF-α ELISA kit, mouse IL-6 ELISA kit, mouse IFN-β ELISA kit and mouse IL-12 p70 ELISA kits were obtained from SolarBio (Beijing, China). CAT was purchased from Aladdin. (Hangzhou, China). Tris-Glycine SDS Buffer, Tris-Glycine Transfer Buffer, and TBST were obtained from CW Biotech (Beijing, China). RIPA lysis buffer and pheylmethylsulfonyl fluoride (PMSF) were purchased from Beyotime Biotechnology (Shanghai, China).

**Synthesis of FeSR780**

The synthesis of FeSR780 was based on previous reports^13^. For the synthesis of compound A, 2-thiophenethiol (0.263 g, 2.26 mmol) and 1-methylpiperazine (0.341 g, 3.40 mmol) were mixed in toluene (8 mL) and stirred at 110℃ for a reflux reaction lasting 8 h. After the reaction, the solution was rotary evaporated to obtain the crude product, which was purified by column chromatography using silica gel and dichloromethane as eluents. The final product, compound A, was orange oil.

Synthesis of FeSR780: Compound A (0.2 g and 1.1 mmol) and croconic acid (0.062 g, 0.44 mmol) were dissolved in a mixture of toluene and n-butanol (30 mL, 1:1) and stirred at 110 ℃ for an overnight reflux reaction. After the reaction, the mixture was filtered and dried. It was then redissolved in methanol and purified by column chromatography using a C18 column with methanol and deionized water as the eluent. FeSR780 was obtained as a black powder after solvent removal under vacuum.

Synthesis of FeSR780: FeCl₃·6H₂O (1.48 mg) and SR780 (3.17 mg) were added to methanol (5 mL) and stirred for 2 min at room temperature. In this solution, 100 mg DSPE-PEG2000 was fully dissolved. After removing the solvent using the thin-film dispersion method, ultrapure water (3 mL) was slowly added, and ultrasonic dispersion was performed to obtain FeSR780.

**Preparation of FeSR780@CAT@Mex NPs**

FeSR780@CAT@Mex NPs were developed using a conventional thin-film hydration method. Briefly, lipids with a ratio of DSPC/DSPE-PEG_2000_/Chol of 90:10:4 (mol/mol/mol) were dissolved in chloroform at 20 °C for 30 min. Subsequently, CAT and FeSR780 solutions (diluted in 4 mL of PBS) were used to hydrate the film at 20 °C for 20 min. Finally, FeSR780@CAT@Mex was harvested by ultrasonication and dialysis.
