# Supplementary figures and images for: pH-Activated Nanoplatform Derived from M1 Macrophages’ Exosomes for Photodynamic and Ferroptosis Synergistic Therapy to Augment Cancer Immunotherapy
Source: Biomater Res. 2025 Mar 6;29:0153. doi: 10.34133/bmr.0153 (PMC11883086; doi:10.34133/bmr.0153)

1c


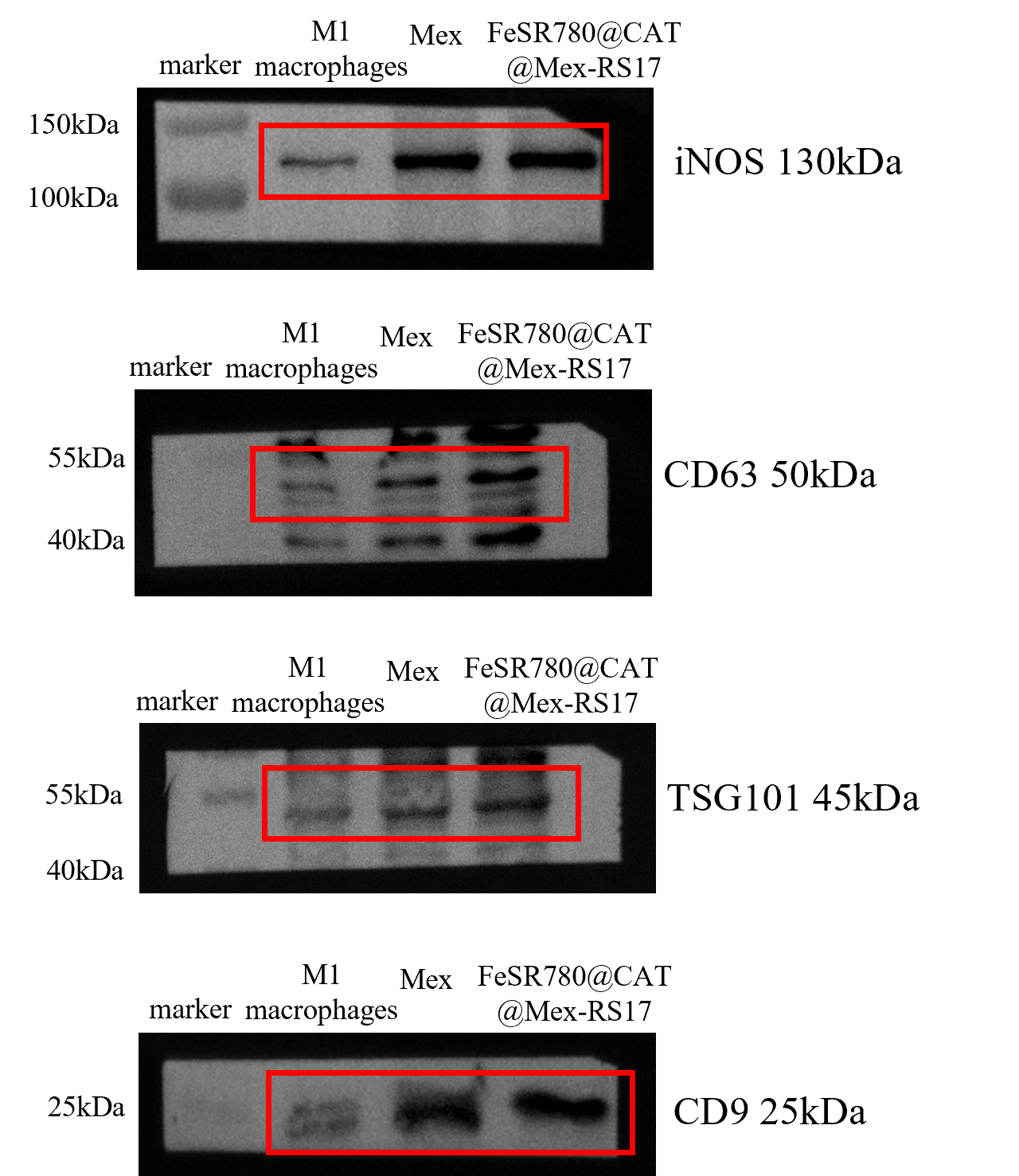


3a


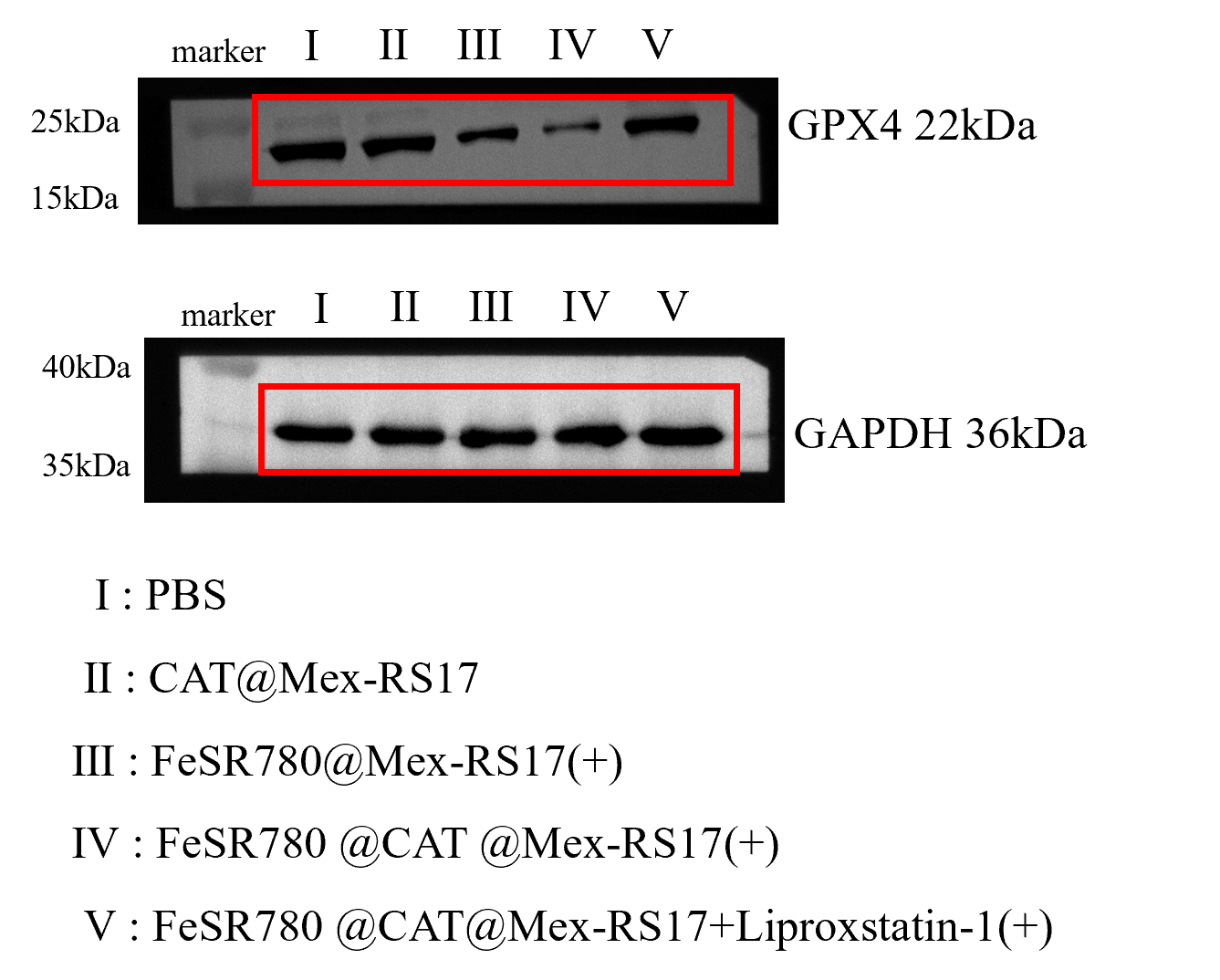


3e


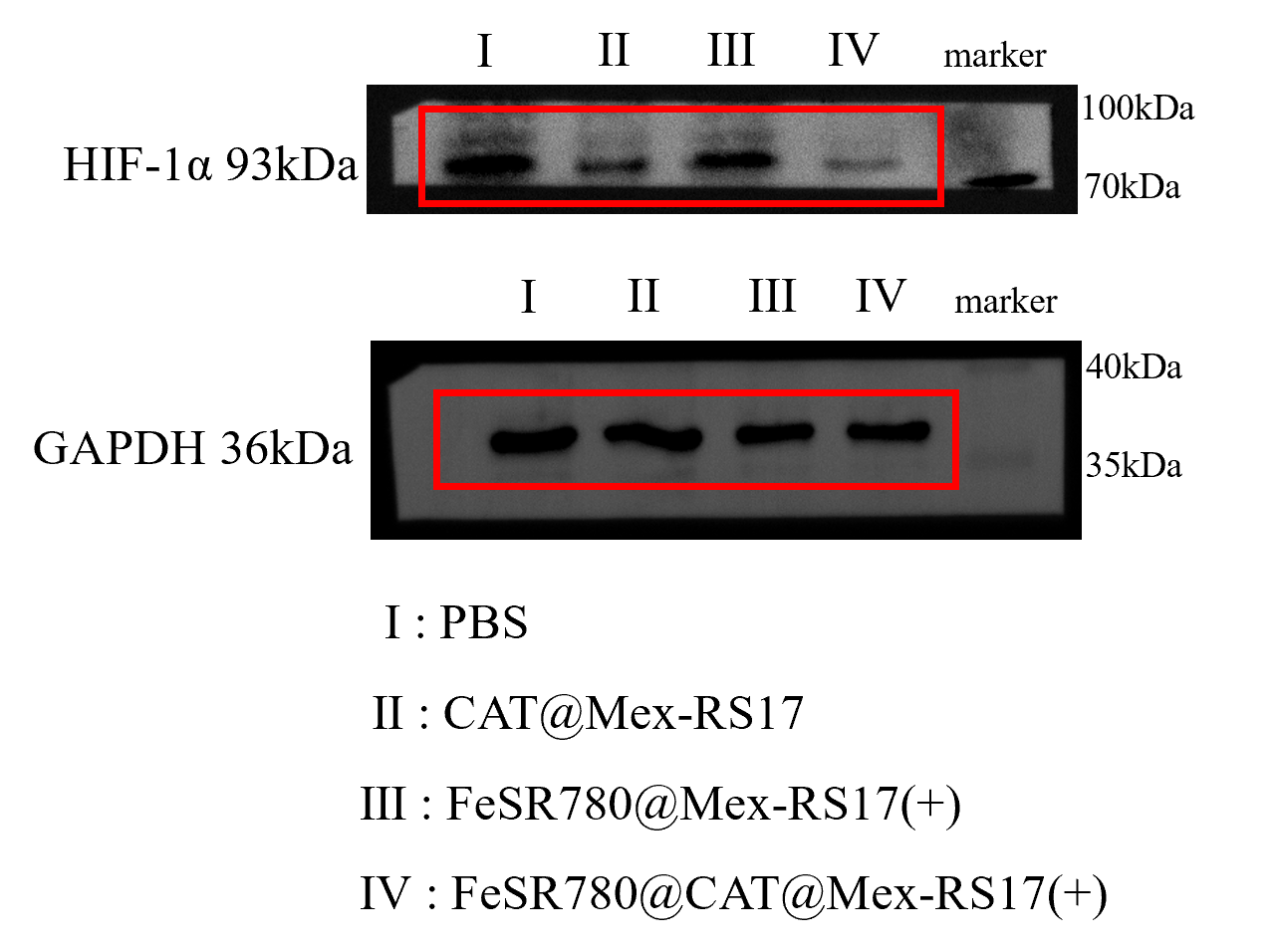


4f


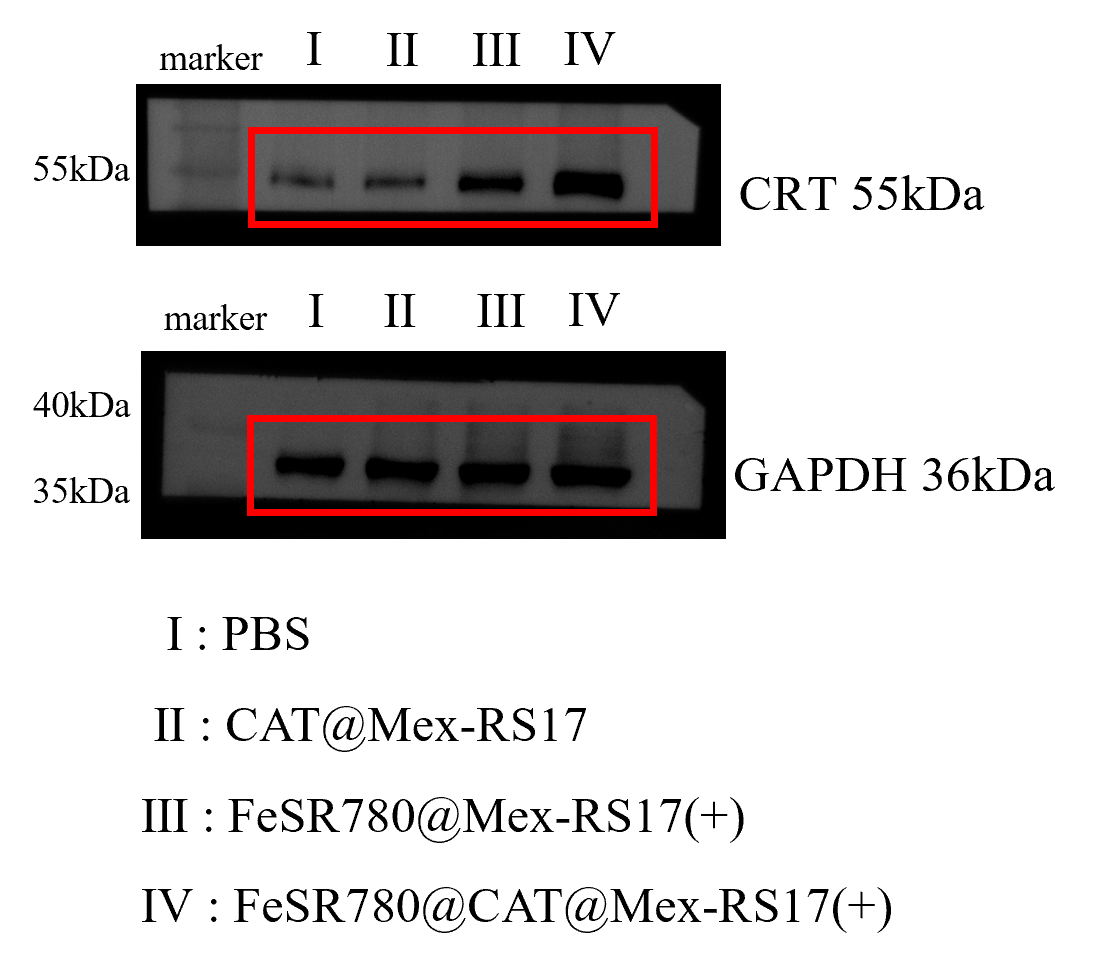


S6


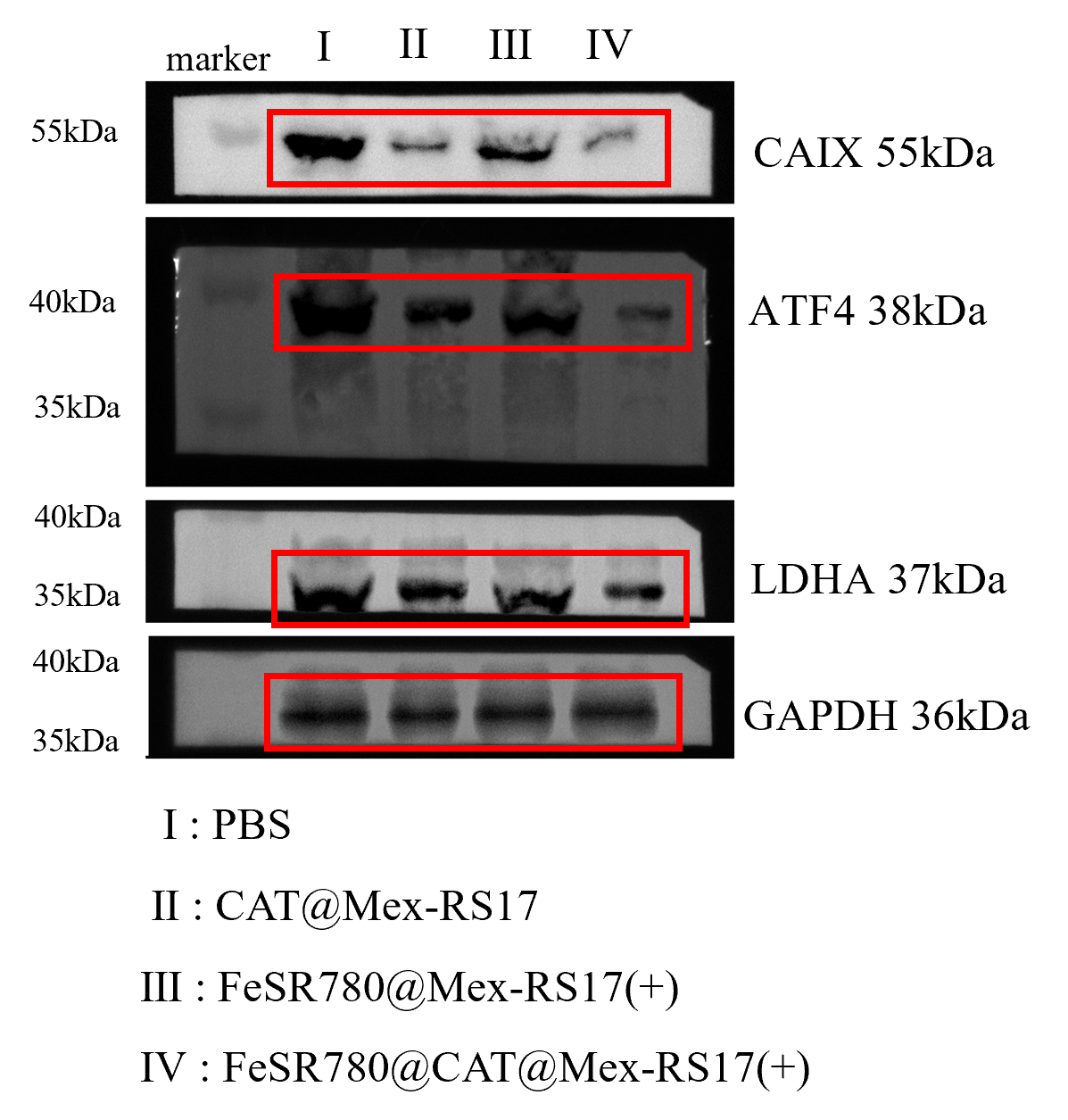

Supplement: Supplementary 1 — Supplementary Text Table S1 Figs. S1 to S7 Western blot bands [file bmr.0153.f1.zip › western blot bands.docx]
